# Clinical remission in severe asthma with biologic therapy: an analysis from the UK Severe Asthma Registry

**DOI:** 10.1183/13993003.00819-2023

**Published:** 2023-12-14

**Authors:** P. Jane McDowell, Ron McDowell, John Busby, M. Chad Eastwood, Pujan H. Patel, David J. Jackson, Adel Mansur, Mitesh Patel, Hassan Burhan, Simon Doe, Rekha Chaudhuri, Robin Gore, James W. Dodd, Deepak Subramanian, Thomas Brown, Liam G. Heaney

**Affiliations:** 1 Wellcome Wolfson Centre for Experimental Medicine, School of Medicine, Dentistry and Biomedical Sciences, Queen's University, Belfast, UK; 2 Belfast Health and Social Care NHS Trust, Belfast, UK; 3 Centre for Public Health, School of Medicine, Dentistry and Biomedical Sciences, Queen's University, Belfast, UK; 4 Royal Brompton and Harefield Hospitals, London, UK; 5 Guy's Severe Asthma Centre, Guy's Hospital, School of Immunology and Microbial Sciences, King's College London, London, UK; 6 University of Birmingham and Heartlands Hospital, Birmingham, UK; 7 Department of Respiratory Medicine, University Hospitals Plymouth NHS Trust, Derriford Hospital, Plymouth, UK; 8 Royal Liverpool University Hospital, Liverpool, UK; 9 The Newcastle upon Tyne Hospitals NHS Foundation Trust, Newcastle upon Tyne, UK; 10 NHS Greater Glasgow and Clyde Health Board, Gartnavel Hospital, Glasgow, UK; 11 Addenbrooke's Hospital, Cambridge University Hospitals NHS Foundation Trust, Cambridge, UK; 12 Academic Respiratory Unit, University of Bristol, Bristol, UK; 13 University Hospitals of Derby and Burton NHS Foundation Trust, Derby, UK; 14 Portsmouth Hospitals NHS Trust, Portsmouth, UK

## Abstract

**Background:**

Novel biologic therapies have revolutionised the management of severe asthma with more ambitious treatment aims. Here we analyse the definition of clinical remission as a suggested treatment goal and consider the characteristics associated with clinical remission in a large, real-world severe asthma cohort.

**Methods:**

This was a retrospective analysis of severe asthma patients registered in the UK Severe Asthma Registry (UKSAR) who met strict national access criteria for biologics. Patients had a pre-biologics baseline assessment and annual review. The primary definition of clinical remission applied included Asthma Control Questionnaire (ACQ)-5 <1.5 and no oral corticosteroids for disease control and forced expiratory volume in 1 s above lower limit of normal or no more than 100 mL less than baseline.

**Results:**

18.3% of patients achieved the primary definition of remission. The adjusted odds of remission on biologic therapy were 7.44 (95% CI 1.73–31.95)-fold higher in patients with type 2 (T2)-high biomarkers. The adjusted odds of remission were lower in patients who were female (OR 0.61, 95% CI 0.45–0.93), obese (OR 0.49, 95% CI 0.24–0.65) or had ACQ-5 ≥1.5 (OR 0.19, 95% CI 0.12–0.31) pre-biologic therapy. The likelihood of remission reduced by 14% (95% CI 0.76–0.97) for every 10-year increase in disease duration. 12–21% of the cohort attained clinical remission depending on the definition applied; most of those who did not achieve remission failed to meet multiple criteria.

**Conclusions:**

18.3% of patients achieved the primary definition of clinical remission. Remission was more likely in T2-high biomarker patients with shorter duration of disease and less comorbidity. Further research on the optimum time to commence biologics in severe asthma is required.

## Tweetable abstract

**Analysis** **of a real-world severe asthma registry shows clinical remission rates of 18%; associated pre-biologic characteristics include male sex, never smoking, BMI <30 kg·m^−2^, shorter disease duration, T2-high biomarkers and lower symptom burden** https://bit.ly/46JLeDb

## Introduction

Asthma is a heterogenous disease of the airways characterised by variable airways hyperresponsiveness and chronic inflammation. It is estimated that 5–10% of asthmatic patients have “severe asthma”, defined by the European Respiratory Society (ERS)/American Thoracic Society (ATS) as “asthma which requires treatment with Global Initiative for Asthma (GINA) step 4–5 treatment or oral corticosteroids (OCS) for 50% of the last year to prevent it becoming uncontrolled” (see supplementary material) [1, 2].

Biologics targeting the type 2 (T2) cytokine pathway have reduced reliance on OCS and revolutionised the management of severe asthma to the extent that there is a need for a defined treatment goal that reflects disease quiescence or stability on treatment [3]. In other chronic inflammatory diseases, such as rheumatoid arthritis and inflammatory bowel disease, this is termed “clinical remission” [4–7].

In severe asthma, a modified Delphi exercise identified core components of disease remission based on the definitions in other chronic inflammatory diseases [8]. The authors identified different targets noting that complete remission (no evidence of T2 biomarkers, absence of symptoms and no corticosteroid exposure) was unlikely to be an achievable goal in severe asthma, but that clinical remission on treatment was a “pragmatic, valuable goal” [8]. The Delphi exercise defined clinical remission as a multicomponent outcome, including 1) no use of systemic corticosteroids for exacerbation or disease control, 2) absence of significant symptoms using a validated instrument, 3) lung function optimisation/stabilisation and 4) patient/provider agreement that clinical remission has been achieved. Although a useful first step in defining clinical remission, it was recognised that a precise definition of “absence of significant symptoms” and “lung function optimisation and stabilisation” needed further work, and perhaps more importantly, by its own admission, the exercise did not attempt to apply this definition to a patient cohort. Another view has suggested that short-acting β_2_-agonist (SABA) use should be an important part of the definition of remission [9].

Clinical remission in severe asthma is not the same as spontaneous remission, which can occur in mild asthma, or a “cure”, which usually refers to complete remission from all manifestations of the disease, while off all treatment for a prolonged period of time. Clinical remission has been explored in clinical trial populations and real-world cohorts of patients with severe asthma [10–13], although direct comparison of these analyses is difficult given different treatments, variable definitions of clinical remission and heterogeneity of asthma severity within these studies [10–13]. In other inflammatory diseases, the definition of clinical remission has been refined over many years [14–17] and a similar evolution of the definition is anticipated in the future.

The UK Severe Asthma Registry (UKSAR) is a national database of patients with uncontrolled asthma referred to specialist UK severe asthma centres [18]. Biologic access for severe asthma in the UK is restricted on the basis of cost-effectiveness by the National Institute of Clinical Healthcare Excellence (NICE), so only patients on maintenance OCS (mOCS) for disease control or requiring three or more courses of prednisolone per year meet access criteria. The objective of this analysis was to utilise this large, real-world cohort of well-characterised severe asthma patients to examine various definitions of clinical remission and assess the pre-biologic clinical characteristics associated with achieving remission.

## Methods

This retrospective, observational analysis includes severe asthma patients registered in UKSAR from specialist, severe asthma services in the UK since January 2016. All patients were ≥18 years old, met ERS/ATS criteria for severe asthma, met NICE access criteria for biologics (see supplementary material), and had baseline assessment at registration and at least one annual review assessment within 9–24 months. The registry has been described elsewhere [17], and has ethical approval for collecting and storing such data with each patient's written consent (Office of Research Ethics Northern Ireland reference 15/NI/0196). A flow diagram for construction of the cohort is given in supplementary figure S1.

The primary definition of remission was: at annual review, Asthma Control Questionnaire (ACQ)-5 <1.5, and no OCS for disease control (no OCS bursts for exacerbations in the last 12 months) and no mOCS for disease control (OCS ≤5 mg per day for hypothalamic–pituitary–adrenal axis suppression permitted), and forced expiratory volume in 1 s (FEV_1_) above lower limit of normal (LLN) or no more than 100 mL less than baseline, pre-biologics FEV_1_. Sensitivity analyses with different definitions of clinical remission were applied as described in the following Statistical methods section.

### Statistical methods

No formal sample size calculation was performed and all available data from UKSAR were used. Descriptive statistics were used to summarise the cohort. Demographic and clinical characteristics at baseline were compared by remission status at 12 months using the Mann–Whitney U-test, Chi-squared test and Fisher's exact test. Within-patient differences between initial assessment and annual review were compared using the one-sample Mann–Whitney U-test and McNemar's test.

Multivariable associations between baseline demographic/clinical characteristics and remission were estimated using logistic regression, adjusting for time to first review and hospital. We chose this limited set of adjustment variables to prevent overadjustment bias, in which adjustment is made for variables that lie on the causal path between the exposure and outcome [19, 20]. Model coefficients were converted to adjusted probabilities of remission for each category with confounders fixed at their mean values; both marginal probabilities and odds ratios are reported. Supplementary analyses additionally adjusted for baseline ACQ-5 and baseline exacerbations. Discrimination for the full model (including all explanatory variables) was assessed using a receiver operating characteristic (ROC) curve and the discriminatory performance was quantified using the area under the curve (AUC).

Where analysis includes T2 biomarker values, blood eosinophil count (BEC) ≥0.15×10^9^ and fractional exhaled nitric oxide (*F*_ENO_) ≥20 ppb are used to describe evidence of T2 inflammation in keeping with GINA guidelines [21]. Obesity was defined as body mass index (BMI) ≥30 kg·m^−2^.

Sensitivity analyses were undertaken to examine the impact of different definitions of clinical remission: 1) ACQ-5 <1.5, and no OCS for disease control (including no OCS-requiring asthma exacerbations and no daily mOCS for asthma), and FEV_1_ above LLN or no more than 100 mL less than baseline, pre-biologics FEV_1_ (primary definition); 2) ACQ-5 <1.5, and no OCS for disease control, and FEV_1_ above LLN or no more than 5% lower than at pre-biologics assessment; 3) ACQ-5 <1.5, and no OCS for disease control, and FEV_1_ above LLN or no lower than at pre-biologics assessment; 4) ACQ-5 <1.5, and no OCS for disease control; 5) ACQ-5 <1.5, and no OCS for disease control, and no SABA; 6) ACQ-5 <1.5, and no OCS for disease control, and ≤2 puffs of SABA per day; and 7) ACQ-5 ≤0.75, and no OCS for disease control, and FEV_1_ above LLN or no more than 100 mL less than baseline, pre-biologics FEV_1_.

Stata version 16.0 SE (StataCorp, College Station, TX, USA) was used to perform the analyses using a complete-case framework.

## Results

A total of 1111 patients met study inclusion criteria across 14 specialist centres (supplementary figure S1). The baseline demographic and clinical details for the entire cohort are outlined in table 1 (data completeness and scalar variables: supplementary tables S1–S3). Of this cohort, 830 had all explanatory variables for the primary definition of remission; their baseline demographics are displayed in supplementary table S4 (data completeness and scalar variables: supplementary tables S5 and S6). This cohort had substantial OCS exposure, a high exacerbation count, T2-high biomarkers and a high symptom burden. Supplementary table S7 outlines changes in outcomes from commencing biologics to 1-year review with significant clinical improvements in exacerbations, acute care utilisation, FEV_1_ and reported symptom burden.

**TABLE 1:** Baseline demographics of the whole cohort at initial assessment prior to commencing biologics (n=1111)

	**n**	**Category**	**Result**
**Time to first annual review (years)**	1111		1.1 (1.0–1.3)
**Sex**	1111	Female	667 (60.0)
**Age at first assessment (years)**	1111		52.0 (41.0–61.0)
**Ethnicity**	1109	White	966 (87.1)
**Smoking status**	1083	Never	723 (66.8)
**BMI (kg·m^−2^)**	1099		29.8 (26.1–34.8)
**Atopic disease**	1111		567 (51.0)
**Depression/anxiety**	1111		129 (11.6)
**Gastro-oesophageal reflux**	1111		224 (20.2)
**Nasal polyps**	1111		237 (21.3)
**OCS bursts for exacerbation (last year)**	1086		5 (3–8)
**Any OCS bursts (last year)**	1086		1020 (93.9)
**Invasive ventilations (ever)**	1058		101 (9.5)
**Any ED attendance for asthma (last year)**	1073		435 (40.5)
**Hospital admissions for asthma (last year)**	1081		431 (39.9)
**Highest BEC recorded (×10^9^ L^−1^)^#^**	1093		0.70 (0.44–1.10)
***F*_ENO_ (ppb)**	831		43.0 (24.0–75.0)
**Composite T2 biomarker group**	811	BEC low (<0.15)/*F*_ENO_ low (<20)	48 (5.9)
		BEC high (≥0.15)/*F*_ENO_ low (<20)	111 (13.7)
		BEC low (<0.15)/*F*_ENO_ high (≥20)	126 (15.5)
		BEC high (≥0.15)/*F*_ENO_ high (≥20)	526 (64.9)
**FEV_1_ (L)**	1089		2.0 (1.5–2.6)
**FEV_1_ (% pred)**	1077		66.9 (52.1–81.5)
**FVC (L)**	1062		3.1 (2.5–3.9)
**FVC (% pred)**	1026		85.9 (73.4–97.8)
**FEV_1_/FVC**	1062		63.6 (53.7–72.1)
**ACQ-5 score**	954		3.2 (2.0–4.0)
**Uncontrolled asthma**	954	ACQ-5 ≥1.5	807 (84.6)
**Maintenance OCS**	1106		638 (57.7)
**Maintenance OCS (mg)^¶^**	1102		10 (8–15)
**ICS**	1111		1103 (99.3)
**ICS dose (µg BDP-equivalent)^+^**	1025		2000 (1600–2000)
**Theophylline**	1099		299 (27.2)
**SABA**	1099		1051 (95.6)
**LABA**	1094		1013 (92.6)
**LAMA**	1095		709 (64.7)
**LTRA**	1059		548 (51.7)
**Maintenance macrolides**	1087		100 (9.2)
**Nebuliser use**	1092		241 (22.1)
**Biologic type commenced**	981	Anti-IL-5	828 (84.4)
		Anti-IgE	150 (15.3)
		Anti-IL-4 receptor α	3 (0.3)
**ERS/ATS severe asthma [1** **]**	1111	Yes	1111 (100.0)

Results are presented as median (interquartile range) or n (%). “Last year” refers to measures assessed using clinical records in the 12 months prior to initial presentation at the severe asthma centre; other measures were as observed or recorded at initial presentation unless otherwise indicated. BMI: body mass index; OCS: oral corticosteroids; ED: emergency department; BEC: blood eosinophil count; *F*_ENO_: fractional exhaled nitric oxide; T2: type 2; FEV_1_: forced expiratory volume in 1 s; FVC: forced vital capacity; ACQ: Asthma Control Questionnaire; ICS: inhaled corticosteroids; BDP: beclometasone dipropionate; SABA: short-acting β_2_-agonists; LABA: long-acting β_2_-agonists; LAMA: long-acting muscarinic antagonists; LTRA: leukotriene receptor antagonists; IL: interleukin; ERS: European Respiratory Society; ATS: American Thoracic Society. ^#^: highest recorded according to prior medical records; ^¶^: among patients on OCS; ^+^: among patients on ICS.

### Baseline characteristics of remission: definition ACQ-5 <1.5, and no OCS for disease control or exacerbation, and FEV_1_ >LLN or ≤ −100 mL from baseline

In total, 18.3% (152/830) (95% CI 15.7–21.1%) of patients met the primary definition for clinical remission after 1 year of biologic therapy. Remission was more common in males (males 22% (75/341) *versus* females 15.7% (77/489); p=0.022), those who were slightly older commencing biologics (55 (48–65) *versus* 51 (41–59) years; p<0.001) and had a shorter duration of asthma (20 (8–32) *versus* 25 (12–37) years; p=0.008) (table 2). Remission was associated with never smoking, nasal polyps, White ethnicity and a lower BMI. The remission cohort was more likely to be composite T2-high (BEC high/*F*_ENO_ high) prior to commencing biologics, with both higher *F*_ENO_ and highest recorded BEC (table 2 and supplementary tables S4–S6).

**TABLE 2:** Baseline pre-biologics characteristics of those who meet remission *versus* those who do not meet remission at annual review (n=830) (remission definition: Asthma Control Questionnaire (ACQ)-5 <1.5, and no maintenance oral corticosteroids (mOCS) or OCS bursts, and forced expiratory volume in 1 s (FEV_1_) above lower limit of normal or ≤100 mL less than pre-biologics FEV_1_)

	**n**	**Category**	**Non-remission** **(81.7% (n=678))**	**Remission** **(18.3% (n=152))**	**p-value**
**Time to first annual review (years)**	830		1.1 (1.0–1.4)	1.1 (1.0–1.2)	0.097
**Sex**	830	Female	412 (60.8)	77 (50.7)	0.022
**Age at first assessment (years)**	830		51.0 (41.0–59.0)	55.0 (48.0–65.0)	<0.001
**Age at onset of symptoms (years)**	737		20.0 (6.0–39.0)	32.0 (14.0–52.0)	<0.001
**Duration of symptoms from baseline (years)**	737		24.5 (12.0–37.0)	20.0 (8.0–32.0)	0.008
**Ethnicity**	828	White	583 (86.2)	141 (92.8)	0.028
**Smoking status**	809	Never	430 (64.9)	109 (74.7)	0.023
**BMI (kg·m^−2^)**	823		30.5 (26.6–35.1)	27.9 (25.4–31.8)	0.001
**Atopic disease**	830		355 (52.4)	72 (47.4)	0.226
**Depression/anxiety**	830		85 (12.5)	3 (2.0)	<0.001
**Gastro-oesophageal reflux**	830		119 (17.6)	25 (16.4)	0.745
**Nasal polyps**	830		123 (18.1)	48 (31.6)	<0.001
**OCS bursts for exacerbation (last year)**	817		5 (3–8)	4 (3–6)	<0.001
**Any OCS bursts (last year)**	817		624 (93.6)	138 (92.0)	0.493
**Frequent exacerbator at baseline**	817	Yes (≥3 in last year)	554 (83.1)	114 (76.0)	0.043
**Invasive ventilations (ever)**	798		68 (10.5)	8 (5.4)	0.058
**Any ED attendance for asthma (last year)**	807		259 (39.2)	34 (23.3)	<0.001
**Hospital admissions for asthma (last year** **)**	814		270 (40.5)	45 (30.4)	0.022
**Highest BEC recorded (×10^9^ L^−1^)** ^#^	821		0.68 (0.40–1.00)	0.79 (0.58–1.33)	<0.001
***F*_ENO_ (ppb)**	638		41.0 (22.0–72.0)	51.0 (35.0–81.0)	0.002
**Composite T2 biomarker group**	627	BEC low (<0.15)/*F*_ENO_ low (<20)	38 (7.7)	2 (1.5)	0.004
		BEC high (≥0.15)/*F*_ENO_ low (<20)	69 (13.9)	10 (7.6)	
		BEC low (<0.15)/*F*_ENO_ high (≥20)	86 (17.3)	21 (16.0)	
		BEC high (≥0.15)/*F*_ENO_ high (≥20)	303 (61.1)	98 (74.8)	
**FEV_1_ (L)**	818		2.0 (1.5–2.6)	2.1 (1.6–2.7)	0.073
**FEV_1_ (% pred)**	811		67.0 (52.4–81.2)	68.6 (55.0–88.2)	0.027
**FVC (L)**	802		3.1 (2.5–3.9)	3.3 (2.7–4.2)	0.048
**FVC (% pred)**	772		85.2 (73.8–97.0)	86.7 (75.8–99.4)	0.065
**FEV_1_/FVC**	802		63.7 (54.3–72.2)	64.5 (54.4–73.6)	0.498
**ACQ-5 score**	736		3.2 (2.2–4.2)	2.0 (1.2–3.4)	<0.001
**Uncontrolled asthma**	711	ACQ-5 ≥1.5	531 (88.8)	91 (65.9)	<0.001
**EuroQoL health scale**	350		60.0 (40.0–70.0)	75.0 (50.0–85.0)	<0.001
**EuroQoL utility**	362		0.7 (0.5–0.8)	0.9 (0.8–1.0)	<0.001
**Maintenance OCS**	826		398 (59.1)	77 (50.7)	0.059
**Maintenance OCS (mg)** ^¶^	474		10 (8–15)	10 (7–10)	0.001
**ICS**	830		676 (99.7)	151 (99.3)	0.500
**ICS dose (µg BDP-equivalent)^+^**	775		2000 (1600–2000)	2000 (1600–2000)	0.556
**Theophylline**	821		184 (27.3)	32 (21.6)	0.153
**SABA**	823		643 (95.5)	142 (94.7)	0.644
**LABA**	818		619 (92.8)	144 (95.4)	0.258
**LAMA**	821		420 (62.7)	105 (69.5)	0.113
**LTRA**	799		327 (50.1)	80 (54.8)	0.303
**Maintenance macrolides**	816		60 (9.0)	9 (6.0)	0.202
**Nebuliser use**	819		164 (24.5)	16 (10.7)	<0.001

Results are presented as median (interquartile range) or n (%). “Last year” refers to measures assessed using clinical records in the 12 months prior to initial presentation at the severe asthma centre; other measures were as observed or recorded at initial presentation unless otherwise indicated. BMI: body mass index; ED: emergency department; BEC: blood eosinophil count; *F*_ENO_: fractional exhaled nitric oxide; T2: type 2; FVC: forced vital capacity; ICS: inhaled corticosteroids; BDP: beclometasone dipropionate; SABA: short-acting β_2_-agonists; LABA: long-acting β_2_-agonists; LAMA: long-acting muscarinic antagonists; LTRA: leukotriene receptor antagonists. ^#^: highest recorded according to prior medical records; ^¶^: among patients on OCS; ^+^: among patients on ICS.

The non-remission cohort had a greater incidence of depression/anxiety (13% *versus* 2%; p<0.001), higher number of exacerbations (5 (3–8) *versus* 4 (3–6); p<0.001), emergency department attendances (39% *versus* 23%; p<0.001) and hospital admissions (41% *versus* 30%; p=0.004). FEV_1_ % pred was slightly lower in the non-remission cohort, although FEV_1_/forced vital capacity ratio was no different. Baseline symptom burden was greater (ACQ-5 3.2 (2.2–4.2) *versus* 2.0 (1.2–3.4); p<0.001), quality of life more impaired and a trend towards increased mOCS use was seen in those who did not achieve remission (table 2 and supplementary table S8).

**FIGURE 1:**
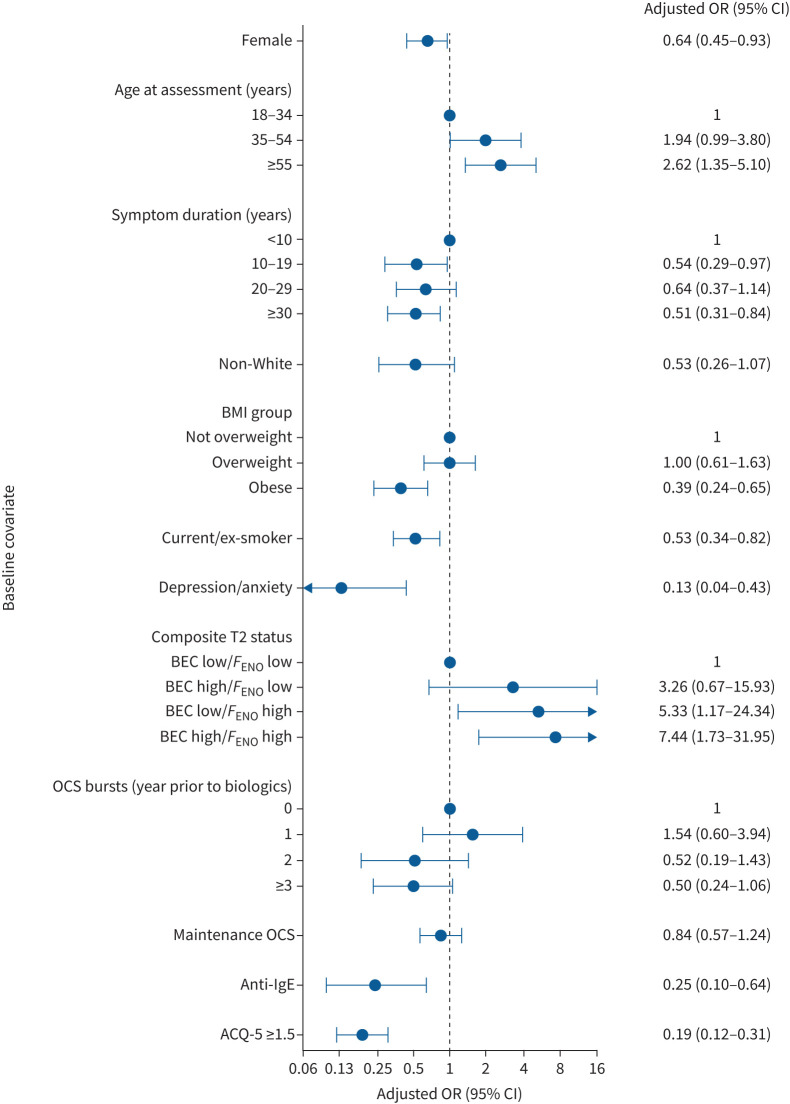
Forest plot of variables associated with remission included in the regression analysis, adjusted for time to follow-up and hospital site. Remission definition: Asthma Control Questionnaire (ACQ)-5 <1.5, and no maintenance oral corticosteroids (mOCS) or OCS bursts, and forced expiratory volume in 1 s (FEV_1_) above lower limit of normal or ≤100 mL less than pre-biologics FEV_1_. BMI: body mass index; T2: type 2; BEC: blood eosinophil count; *F*_ENO_: fractional exhaled nitric oxide.

There were distinct differences between the group of patients that achieved remission and those that did not; age commencing biologics, obesity, smoking status, composite T2 status, comorbid depression/anxiety and ACQ-5 continue to be significantly different between the cohorts when adjusting for potential confounders including hospital site and time to review (figure 1 and supplementary table S9).

Clinical remission was more likely with a shorter duration of symptoms; every increasing 10 years duration of asthma symptoms before commencing biologics was associated with a 14% decreased odds of achieving remission (adjusted OR 0.86, 95% CI 0.76–0.97; p=0.013), such that an individual commencing biologics with over 30 years of asthma symptoms is 49% less likely to achieve remission than someone with a duration of symptoms of less than 10 years (adjusted OR 0.51, 95% CI 0.31–0.84; p=0.008). Being older when commencing biologics was associated with increased remission; with every increasing 10 years of age at pre-biologics assessment, the odds of remission increased by 31% (adjusted OR 1.31, 95% CI 1.15–1.50; p<0.001). The odds of remission for those who were composite T2-high (BEC high/*F*_ENO_ high) was 7.44 times as high as the composite T2-low group (adjusted OR 7.44, 95% CI 1.73–31.95; p=0.007) (figure 1 and supplementary table S9).

Females were 36% less likely to achieve remission (adjusted OR 0.64, 0.45–0.93; p=0.018). The odds of remission were 47% lower in obese compared with non-obese patients (adjusted OR 0.53, 95% CI 0.34–0.82; p=0.004) and with every increased unit of BMI ≥30 kg·m^−2^ a patient was 5% less likely to achieve remission (adjusted OR 0.95, 95% CI 0.92–0.98; p<0.001). Comorbid depression/anxiety (adjusted OR 0.13, 95% CI 0.04–0.43; p=0.001) and a higher pre-biologics symptom burden (ACQ-5 ≥1.5: adjusted OR 0.19, 95% CI 0.12–0.31; p<0.001) were associated with a significantly lower odds of remission. Anti-IgE therapy was associated with 81% lower odds of remission than anti-interleukin-5 biologics (note that access criteria and thereby the patient population receiving these therapies are different) (figure 1 and supplementary table S9). Ethnic disparities lose significance when adjusted for hospital site and baseline morbidity (adjusted OR 0.53, 95% CI 0.26–1.07; p=0.077) (figure 1 and supplementary table S9). The adjusted probabilities of achieving clinical remission for each covariate are plotted in supplementary figure S2.

**FIGURE 2:**
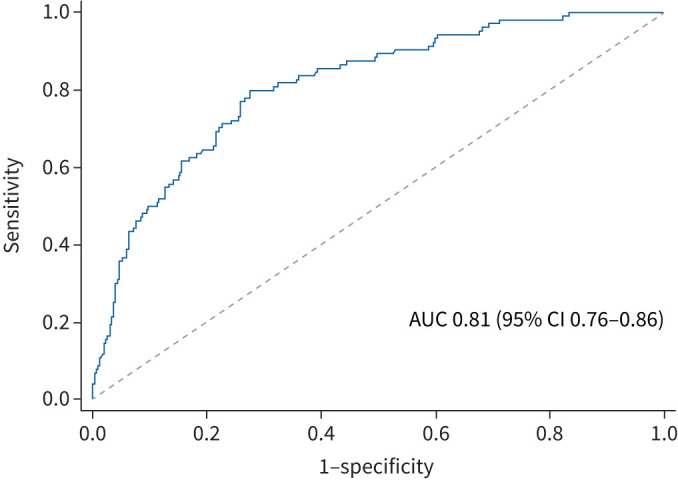
Receiver operating curve for explanatory variables included in the clinical remission model. Variables in the remission model include gender, age at commencing biologics, symptom duration, ethnicity, body mass index, smoking history, depression/anxiety, composite type 2 status, oral corticosteroid (OCS) bursts in the year prior to biologics, maintenance OCS, biologic mechanism, hospital site, time to follow-up and Asthma Control Questionnaire-5. AUC: area under the curve.

**FIGURE 3:**
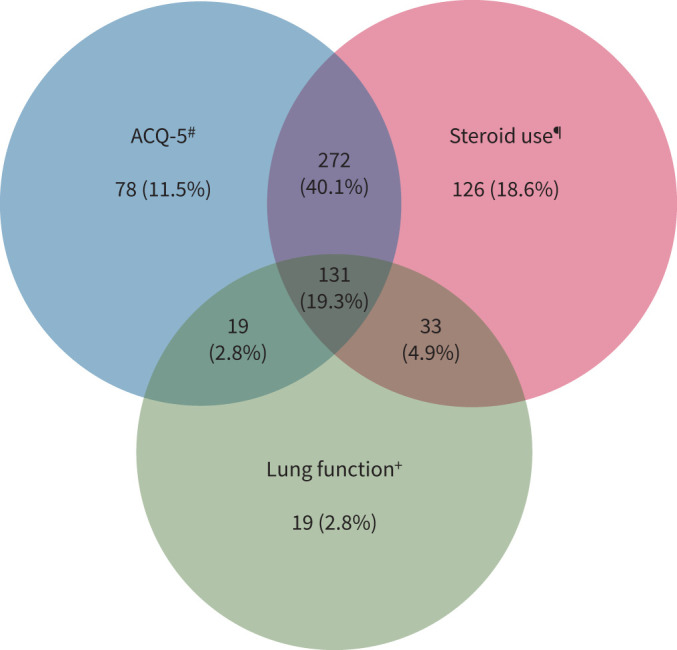
Reasons for not meeting remission criteria after 1 year on biologic therapy. 30% (202) failed to meet lung function criteria, 74% (500) failed to meet Asthma Control Questionnaire (ACQ)-5 criteria and 83% (562) failed to meet steroid criteria; 67% of those who did not achieve remission failed to meet at least two of the remission criterion. ^#^: ACQ-5 ≥1.5; ^¶^: at least one exacerbation and/or maintenance oral corticosteroids >5 mg; ^+^: forced expiratory volume in 1 s below lower limit of normal and >100 mL less than baseline.

A ROC suggests good discrimination of the full model predicting remission (AUC 0.81, 95% CI 0.76–0.86) (figure 2), with ACQ-5 being the best prognostic marker (supplementary table S10). Most of those who did not meet the criteria for remission failed to meet a number of individual remission criteria (figure 3).

### Remission: definition ACQ-5 <1.5, and no OCS for disease control, and FEV_1_ >LLN or ≥ −5% FEV_1_ reduction from baseline

Using a proportional reduction in FEV_1_ (5%), rather than an actual volume reduction (100 mL), in the definition of remission did not affect the rates of remission (18.3% (152/830) (95% CI 15.7–21.1%)). This definition did not change the baseline characteristics associated with remission and non-remission (supplementary table S11 and supplementary figure S3).

### Remission: definition ACQ-5 <1.5, and no OCS for disease control, and FEV_1_ >LLN or ≥baseline FEV_1_

Applying a stricter FEV_1_ criteria for remission (FEV_1_ no lower than baseline) had minimal impact on the remission analysis (17.7% (147/830) (95% CI 15.2–20.4%) achieved remission), and did not substantially change the characteristic differences between the remission and non-remission cohorts (supplementary table S12 and supplementary figure S4).

### Remission: definition ACQ-5 <1.5, and no OCS for disease control

A definition of remission which included only two criteria for remission and did not have any lung function criteria resulted in a slightly higher rate of remission (21.2% (196/925) (95% CI 18.6–24.0%)). Compared with analyses including FEV_1_ in the remission definition, this definition had no difference in outcomes in ethnicity (p=0.060) (supplementary table S13 and supplementary figure S5).

### Remission: definition ACQ-5 <1.5, and no OCS for disease control, and no SABA

Only 13.8% (123/891) (95% CI 11.6–16.2%) of the cohort reached remission using this criterion, of which 75% (667/891) did not meet the SABA criteria, 67% (594/891) did not meet the OCS criteria and 60% (533/891) did not meet the ACQ-5 criteria. In this analysis, compared with the primary definition of remission, the difference in gender, ethnicity, atopic disease and composite T2 biomarker groups was lost between those who met the difference in remission and those who did not (supplementary table S14 and supplementary figure S6).

### Remission: definition ACQ-5 ≤0.75, and no OCS for disease control, and FEV_1_ >LLN or ≤ −100 mL from baseline

When applying a definition of remission which includes more stringent asthma control (ACQ-5 ≤0.75, no mOCS or bursts for exacerbations and FEV_1_ ≤100 mL from baseline or >LLN), only 11.9% (99/830) (95% CI 9.8–14.3%) met the definition of remission (supplementary table S16 and supplementary figure S8)

## Discussion

In this analysis of patients with severe asthma from a large national registry, 18% met the primary definition of remission, with distinct clinical and demographic characteristics seen in the remission *versus* non-remission cohorts.

At baseline, this cohort was typical of a severe asthma patient population and had considerable corticosteroid use with elevated T2 biomarkers, a high exacerbation rate, impaired lung function and a high symptom burden. With use of biologic therapy, after 1 year, acute exacerbations and emergency healthcare utilisation were substantially reduced with improved patient-reported outcome measures (PROMs); however, only a minority of patients achieved clinical remission.

Clinical remission was more commonly achieved in males, never-smokers and non-obese individuals with higher T2 biomarkers. These patients were older at disease onset, with shorter disease duration, lower symptom score and fewer exacerbations at initiation of biologic therapy.

Non-remission was more common with earlier asthma onset and longer disease duration prior to commencing biologics, with greater exacerbation burden and resultant OCS exposure. Asthma exacerbations are associated with accelerated lung function decline compared with aged-matched peers (healthy controls and non-exacerbating asthmatic subjects), with the effect of multiple exacerbations being cumulative, and we see lung function was lower in the non-remission cohort with a higher exacerbation burden [22]. Further, there is evidence for OCS toxicities at relatively low exposure, increasing with cumulative dose, and a greater risk of comorbidities in young adults compared with age-matched non-OCS exposed controls [23–25]. Non-remission was associated with female sex, obesity, comorbid depression/anxiety and frequent exacerbations, and these factors have previously been associated with poor asthma outcomes in non-biologic severe asthma cohorts [26–28]. It is recognised that comorbid conditions such as obesity, depression/anxiety and breathing pattern disorder can cause persistent symptoms in this population which are captured on asthma control PROMs [26, 29, 30]. While biologics can effectively address T2 inflammation, they are unlikely to address symptoms driven by these non-T2 related comorbidities, which require a different, multidisciplinary team approach [29]. Patients who achieved clinical remission had higher T2 biomarkers, which is in keeping with previous findings that these predict a better response to biologics [3, 31–35]. Taken together, the data suggest that introduction of biologics earlier in the disease course, before comorbid disease associated with OCS exposure accumulates, may make asthma remission a more realistic target for future generations of severe asthma patients.

The described clinical and demographic features associated with remission are present at initial assessment and the ROC analysis confirms that together the variables included in the regression model are highly discriminatory for predicting remission; however, no single feature was able to predict remission. Recognition of these clinical and demographic characteristics at baseline will help clinicians identify patients who are more likely to achieve clinical remission, and importantly to inform patient discussion about what can realistically be achieved with biologic treatment.

The criteria for clinical remission on treatment have been the subject of a series of consensus statements [8, 9, 36], but none of these have been prospectively validated to show that achieving clinical remission improves long-term clinical outcome. An essential criterion of all these statements is the removal of “OCS for exacerbations and disease control”. In this cohort, there was an overall substantial reduction in exacerbations (median (interquartile range) 5 (3–8) *versus* 1 (0–3)) after 1 year of biologics; however, despite this substantial impact, many patients failed to reach remission criteria because of ongoing OCS use.

Another criterion is the “absence of significant symptoms”, which is challenging as severe asthma patients are often highly symptomatic [18, 37] and as discussed earlier, symptoms may be due to non-asthma comorbidity. Applying a “controlled asthma” cut-point of ACQ-5 ≤0.75 [38] sets an ambitious target for most severe asthma patients and was only achieved in 21% (178/830) of this cohort. However, even when using a threshold of ACQ-5 <1.5 (the threshold for uncontrolled asthma [38]) only 40% (330/830) achieved this target. The cut-point for symptom control in the definition of remission will be part of the evolving discussion on this topic, and a recent consensus statement suggested remission should be reserved for an ACQ <0.75 and reliever use once or less per month [39]. Accepting that a good clinical response is not the same as clinical remission, it therefore may not be appropriate to use an ACQ-5 cut-point of <1.5 for this definition. Patient input and evaluation of symptom burden in patients where biologics have been introduced earlier with a shorter duration of disease and less OCS exposure will be useful in considering this matter in the future.

Lung function criterion is not straightforward to consider longitudinally in severe asthma; FEV_1_ measurement is influenced by natural daily variation, bronchodilator medication and natural lung function decline which varies with gender (∼30 mL per year in healthy females and ∼43 mL per year in healthy males) [40, 41]. Additionally, there is no good evidence of what constitutes “stable lung function” over time in severe asthma. This analysis demonstrates that using absolute value reduction of −100 mL, proportional reduction of −5% or baseline FEV_1_ as a cut-point for the definition of remission does not substantially affect the proportion of patients achieving remission, or significantly change the clinical characteristics of the remission/non-remission groups. Lung function assessment in biologic-treated severe asthma cohorts will require longitudinal assessment over years to draw evidence-based conclusions on what constitutes “lung function stability” and whether the use of age-adjusted lung function equations is sufficient [42].

In terms of the final criterion, specifically “patient/clinician agreement on the achievement of remission”, information on this issue was not available in this retrospective analysis. It will be important to explore this prospectively in future studies as shared decision making and agreement is an important aspect of clinical remission in other inflammatory diseases [43–45].

Advantages of this analysis are its size and the “real-world” nature of the cohort. Limitations include the possibility of type 1 error due to testing across multiple variables within the cohort; however, this is a hypothesis-generating study and logistic regression modelling was limited to a smaller number of variables. As with all “real-world” observational studies, there is an inherent possibility that the proportion of patients achieving remission may be overestimated due to regression to the mean, but it would be unethical to have a control arm for comparison [45]. Lastly, longitudinal data are needed to assess if clinical remission is maintained over time in severe asthma patients on biologics and whether this improves clinical outcomes.

In summary, this severe asthma cohort benefited substantially from biologic therapy, although only 18% of patients achieved clinical remission using the proposed definition. The characteristics of those who attain remission and those who do not are described, with the suggestion that remission is achieved in patients with shorter duration of disease, fewer comorbidities and greater T2 inflammation. Further studies are needed to demonstrate that achievement and maintenance of disease remission improve long-term clinical outcomes in severe asthma and specifically whether earlier intervention with effective biologic treatment can improve long-term disease trajectory.

## Supplementary material

10.1183/13993003.00819-2023.Supp1 **Please note:** supplementary material is not edited by the Editorial Office, and is uploaded as it has been supplied by the author.
Supplementary material ERJ-00819-2023.Supplement

## Shareable PDF

10.1183/13993003.00819-2023.Shareable1 This one-page PDF can be shared freely online.
Shareable PDF ERJ-00819-2023.Shareable
