# Supplementary material for: Clinical remission in severe asthma with biologic therapy: an analysis from the UK Severe Asthma Registry
Source: Eur Respir J. 2023 Dec 14;62(6):2300819. doi: 10.1183/13993003.00819-2023 (PMC10719453; doi:10.1183/13993003.00819-2023)
Supplement: Supplementary file 1 [file ERJ-00819-2023.Supplement.pdf]

Clinical remission in severe asthma with Biologic Therapy: an analysis from the UK Severe Asthma Registry: Online Supplement.

Definitions of severe asthma (ATS/ERS and GINA) [E1]

Definition of severe asthma: American Thoracic society/ European Respiratory Society

Severe asthma is defined as asthma which requires GINA step 4–5 asthma treatment (high dose inhaled corticosteroid (ICS) and long acting Beta-agonist (LABA) or leukotriene modifier/theophylline) and/or systemic corticosteroids (OCS) for 50% of the previous year to prevent it from becoming ‘‘uncontrolled’’ or which remains ‘‘uncontrolled‘‘ despite this therapy.
> Uncontrolled asthma is defined as at least one of the following:

1. Poor symptom control: Asthma Control Questionnaire (ACQ) consistently ≥1.5, Asthma Control Test (ACT) <20 (or ‘‘not well controlled’’ by NAEPP/GINA guidelines)
2. Frequent severe exacerbations: two or more bursts of systemic OCS (3 days each) in the previous year
3. Serious exacerbations: at least one hospitalisation, ICU stay or mechanical ventilation in the previous year
4. Airflow limitation: after appropriate bronchodilator withhold FEV1 ,80% predicted (in the face of reduced FEV1/FVC defined as less than the lower limit of normal)

> Controlled asthma that worsens on tapering of these high doses of ICS or systemic OCS (or additional biologics)

Definition of severe asthma: Global initiative for asthma (GINA) [E2]

Severe asthma is uncontrolled (poor symptom control, ≥ 2 exacerbations per year, requiring OCS, ≥1 severe exacerbation requiring hospitalisation) despite adherence factors with maximal optimized therapy and treatment of contributory factors, or that worsens when high dose treatment is decreased.

Definitions of remission set forth by the ‘expert consensus framework for asthma remission as a treatment goal’.[E3]

Clinical remission: on treatment	Clinical remission: off treatment
**≥12 months:**<br>1) Sustained absence of significant asthma symptoms using validated instrument AND<br>2) Optimisation and stabilization of lung function AND<br>3) Patient & HCP agreement regarding remission AND<br>4) No OCS for exacerbation or disease control	Same criteria but without asthma treatment in ≥12 months
**Complete remission: on treatment**	**Complete remission: off treatment**
1. Meets definition for clinical remission **AND**<br>2. Current, objective evidence of the resolution of previous asthma-related inflammation* **AND**<br>3. In appropriate research settings: current negative bronchial hyperresponsiveness	Same criteria but without asthma treatment in ≥12 months

NICE criteria for commencing biologics in the UK for uncontrolled asthma despite optimisation and assessment of adherence:

Benralizumab:[E4]

Benralizumab, as an add-on therapy, is recommended as an option for treating severe eosinophilic asthma that is inadequately controlled in adults despite maintenance therapy with high-dose inhaled corticosteroids and long-acting beta-agonists, only if:

- the person has agreed to and followed the optimised standard treatment plan and
- the blood eosinophil count has been recorded as 300 cells per microlitre or more and the person has had 4 or more exacerbations needing systemic corticosteroids in the previous 12 months, or has had continuous oral corticosteroids of at least the equivalent of prednisolone 5 mg per day over the previous 6 months (that is, the person is eligible for mepolizumab) or
- he blood eosinophil count has been recorded as 400 cells per microlitre or more with 3 or more exacerbations needing systemic corticosteroids in the past 12 months (that is, the person is eligible for reslizumab).

Dupilumab:[E5]

Dupilumab as add-on maintenance therapy is recommended as an option for treating severe asthma with type 2 inflammation that is inadequately controlled in people 12 years and over, despite maintenance therapy with high-dose inhaled corticosteroids and another maintenance treatment, only if:

- the dosage used is 400 mg initially and then 200 mg subcutaneously every other week
- the person has agreed to and follows an optimised standard treatment plan
- the person has a blood eosinophil count of 150 cells per microlitre or more and fractional exhaled nitric oxide of 25 parts per billion or more, and has had at least 4 or more exacerbations in the previous 12 months
- the person is not eligible for mepolizumab, reslizumab or benralizumab, or has asthma that has not responded adequately to these biological therapies

Mepolizumab:[E6]

Mepolizumab, as an add-on therapy, is recommended as an option for treating severe refractory eosinophilic asthma, only if:

- for adults who have agreed to and followed the optimised standard treatment plan and
- the blood eosinophil count has been recorded as 300 cells per microlitre or more and the person has had at least 4 exacerbations needing systemic corticosteroids in the previous 12 months, or has had continuous oral corticosteroids of at least the equivalent of prednisolone 5 mg per day over the previous 6 months or

- the blood eosinophil count has been recorded as 400 cells per microlitre or more and the person has had at least 3 exacerbations needing systemic corticosteroids in the previous 12 months (so they are also eligible for either benralizumab or reslizumab).

Omalizumab:(E7)

Omalizumab is recommended as an option for treating severe persistent confirmed allergic IgE-mediated asthma as an add-on to optimised standard therapy in people aged 6 years and older:

- who need continuous or frequent treatment with oral corticosteroids (defined as 4 or more courses in the previous year)

Reslizumab:(E8)

Reslizumab, as an add-on therapy, is recommended as an option for the treatment of severe eosinophilic asthma that is inadequately controlled in adults despite maintenance therapy with high-dose inhaled corticosteroids plus another drug, only if:

- the blood eosinophil count has been recorded as 400 cells per microlitre or more
- the person has had 3 or more severe asthma exacerbations needing systemic corticosteroids in the past 12 months and

Assessments:

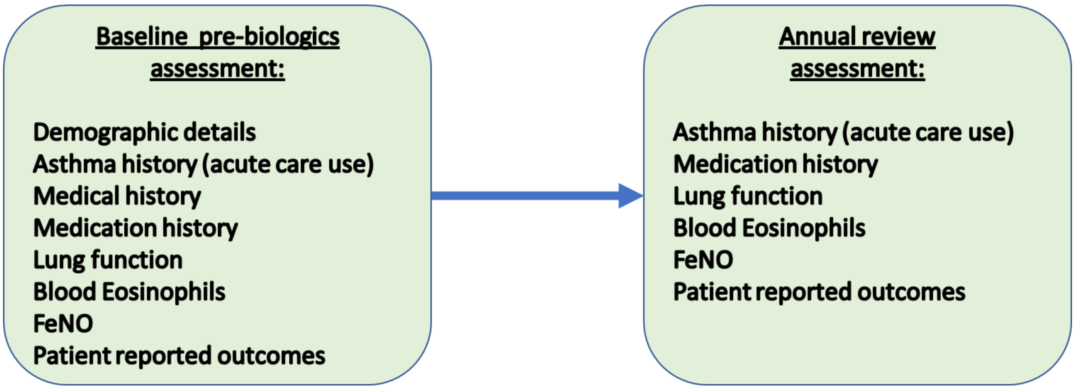

Data recorded at baseline assessment, includes demographic details, asthma/atopy history, comorbidities, exacerbation/acute care attendance in the last 12 months, medication history, biomarkers (blood eosinophil count (BEC) and fractional exhaled nitric oxide (FeNO) and lung function (GLI 2012 predictive values, [E9]). Patient reported outcome measures (PROMs) completed at each assessment included asthma control assessment (ACQ5, asthma control questionnaire and quality of life measures (EuroQOL 5L5D). At annual review, acute care use, medication history,

biomarkers, lung function and PROMS are recorded. To reduce bias towards biologic classes, all biologics approved for use for severe asthma treatment in the UK were included in the analysis, including anti-IL5, anti-IL5R, anti-IL4αr and anti-IgE.

Patient-reported outcome measures (PROMs) in this study addressed change in quality-of-life and asthma severity.

Asthma Control Questionnaire (ACQ5):

Asthma control. Asthma control was assessed by the five item asthma control questionnaire.[E10] An ACQ5 score of 0.75 reflects adequate asthma control, whereas a score of 1.5 or over signals inadequate asthma control. The MCID is a reduction in ACQ5 score of 0.5 or greater.[E11]

EuroQol:

Quality of life. The EuroQoL-5L5D Health scale is a visual analogue scale with which patients report their overall health,[E12] 100 is the best health possible and 0 reflects the worst overall health. The EuroQoL-5L5D Index value reflects impairment of activities of daily living, the closer the score is to 1, the less the impairment of daily living. The change in Index Value following an intervention allows evaluation of the health economics of the intervention through calculation of quality-adjusted life years.

Figure S1: Flow diagram for construction of UKSAR cohort

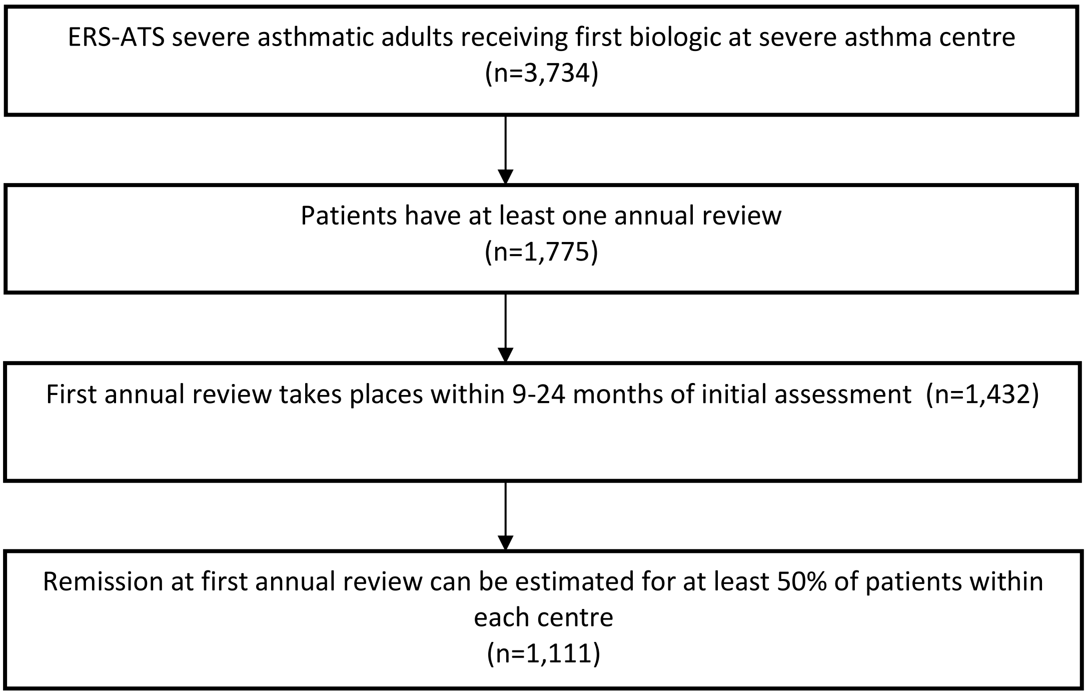

Table S1: Data completeness (whole cohort (n=1,111)

Variable	n (%) non-missing	n (%) missing
**Time until Annual review (Years)**	1,111 (100.0%)	0 (0.0%)
**Sex**	1,111 (100.0%)	0 (0.0%)
**Age At First Assessment (Years)**	1,111 (100.0%)	0 (0.0%)
**Age at Onset of Symptoms (Years)**	976 (87.8%)	135 (12.2%)
**Duration Of Symptoms From Baseline (Years)**	976 (87.8%)	135 (12.2%)
**Ethnicity**	1,109 (99.8%)	2 (0.2%)
**Smoking Status**	1,083 (97.5%)	28 (2.5%)
**BMI (kg-m2)**	1,099 (98.9%)	12 (1.1%)
**Atopic Disease**	1,111 (96.8%)	37 (3.2%)
**Depression or Anxiety**	1,111 (100.0%)	0 (0.0%)
**Gastro-oesophageal Reflux**	1,111 (100.0%)	0 (0.0%)
**Nasal polyps**	1,111 (100.0%)	0 (0.0%)
**OCS bursts (Last Year)**	1,086 (97.7%)	25 (2.3%)
**Frequent exacerbator at baseline**	1,086 (97.7%)	25 (2.3%)
**Invasive Ventilations (Ever)**	1,058 (95.2%)	53 (4.8%)
**ED Attendances For Asthma (Last Year)**	1,073 (96.6%)	38 (3.4%)
**Hospital Admissions For Asthma (Last Year)**	1,081 (97.3%)	30 (2.7%)
**Highest Blood Eosinophil Count ($N/10^9L$) †**	1,093 (98.4%)	18 (1.6%)
**Blood Eosinophil Count ($N/10^9L$)**	1,085 (97.7%)	26 (2.3%)
**FeNO (ppb)**	831 (74.8%)	280 (25.2%)
**Composite Type 2 status**	811 (73.0%)	300 (27.0%)
**FEV_1 (L)**	1,089 (98.0%)	22 (2.0%)
**FEV_1 (% Predicted)**	1,077 (96.9%)	34 (3.1%)
**FVC (L)**	1,062 (95.6%)	49 (4.4%)
**FVC (% Predicted)**	1,026 (92.3%)	85 (7.7%)
**FEV_1/FVC**	1,062 (95.6%)	49 (4.4%)
**ACQ5 Score**	954 (85.9%)	157 (14.1%)
**Uncontrolled asthma**	954 (85.9%)	157 (14.1%)
**Puffs of SABA most days (ACQ Q6)**	924 (83.2%)	187 (16.8%)
**EuroQoL Health Scale**	454 (40.9%)	657 (59.1%)
**EuroQoL Utility**	470 (42.3%)	641 (57.7%)
**Maintenance OCS**	1,106 (99.5%)	5 (0.5%)
**Maintenance OCS (mg) ***	634 (99.4%)	4 (0.6%)
**Inhaled corticosteroids (ICS)**	1,111 (100.0%)	0 (0.0%)
**ICS Dose (BDP equivalent-μg) *****	1,025 (92.9%)	78 (7.1%)
**Theophylline**	1,099 (98.9%)	12 (1.1%)
**Short-acting β2-agonist (SABA)**	1,099 (98.9%)	12 (1.1%)
**Long-acting β2-agonist (LABA)**	1,094 (98.5%)	17 (1.5%)
**Leukotriene Receptor Antagonists**	1,059 (95.3%)	52 (4.7%)
**Maintenance Macrolides**	1,087 (97.8%)	24 (2.2%)
**Nebuliser**	1,092 (98.3%)	19 (1.7%)
**Biologic commenced**	981 (88.3%)	130 (11.7%)
**Biologic type commenced**	981 (88.3%)	130 (11.7%)
**ERS-ATS Severe Asthma**	1,111 (100.0%)	0 (0.0%)
**Hospital**	1,111 (100.0%)	0 (0.0%)

† highest recorded according to prior medical records. 'Last Year' refers to measures assessed using clinical records in the 12 months prior to initial presentation at the severe asthma centre, other measures were as observed or recorded at initial presentation unless otherwise indicated.*among patients on oral corticosteroids**among patients on inhaled corticosteroids

BMI: Body Mass Index; OCS: Oral Corticosteroid Steroids; ED: Emergency Department; FeNO: Fractional Exhaled Nitric Oxide; ppb: Parts per Billion; FEV_1: Forced Expiratory Volume (1 second); FVC: Forced Vital Capacity; ACQ: Asthma Control Questionnaire: ICS: Inhaled Corticosteroid Steroids: BDP: Beclometasone Dipropionate; SABA: Short-Acting Beta Agonists; LABA: Long-Acting Beta Agonists; LAMA: Long-Acting Muscarinic Antagonist; ERS-ATS: European Respiratory Society-American Thoracic Society definition severe asthma 'asthma which requires treatment with GINA step 4-5 treatment or oral corticosteroids (OCS) for 50% of the last year to prevent it becoming uncontrolled'

Table S2: Summary statistics for scalar variables (whole cohort (n=1,111)

Variable	Mean (SD)	Median (IQR)
**Time until Annual review (Years)**	1.2 (0.3)	1.1 (1.0,1.3)
**Age At First Assessment (Years)**	50.8 (14.5)	52.0 (41.0,61.0)
**Age at Onset of Symptoms (Years)**	25.7 (19.9)	21.0 (7.0,41.0)
**Duration Of Symptoms From Baseline (Years)**	25.1 (17.3)	23.0 (10.0,37.0)
**BMI (kg-m2)**	30.8 (7.4)	29.8 (26.1,34.8)
**OCS bursts (Last Year)**	5.9 (4.0)	5 (3,8)
**ED Attendances For Asthma (Last Year)**	1.7 (3.9)	0 (0,2)
**Hospital Admissions For Asthma (Last Year)**	1.1 (2.1)	0 (0,1)
**Highest Blood Eosinophil Count (N/10^9L) †**	0.94 (1.11)	0.70 (0.44,1.10)
**Blood Eosinophil Count (N/10^9L)**	0.50 (0.57)	0.40 (0.20,0.65)
**FeNO (ppb)**	57.8 (49.0)	43.0 (24.0,75.0)
**FEV_1 (L)**	2.1 (0.8)	2.0 (1.5,2.6)
**FEV_1 (% Predicted)**	67.3 (21.3)	66.9 (52.1,81.5)
**FVC (L)**	3.3 (1.0)	3.1 (2.5,3.9)
**FVC (% Predicted)**	85.9 (19.1)	85.9 (73.4,97.8)
**FEV_1/FVC**	63.1 (18.7)	63.6 (53.7,72.1)
**ACQ5 Score**	3.0 (1.4)	3.2 (2.0,4.0)
**EuroQoL Health Scale**	56.5 (21.3)	60.0 (40.0,70.0)
**EuroQoL Utility**	0.7 (0.3)	0.8 (0.6,0.9)
**Maintenance OCS (mg) ***	12.9 (8.7)	10 (8,15)
**ICS Dose (BDP equivalent-µg) ****	1950.5 (640.1)	2000 (1600,2000)

† highest recorded according to prior medical records. 'Last Year' refers to measures assessed using clinical records in the 12 months prior to initial presentation at the severe asthma centre, other measures were as observed or recorded at initial presentation unless otherwise indicated

* among patients on OCS

** among patients on inhaled corticosteroids

SD: Standard Deviation; IQR: InterQuartile Range; BMI: Body Mass Index; ED: Emergency Department; FEV_1: Forced Expiratory Volume in 1 second; FVC: Forced Vital Capacity; FeNO: Fractional exhaled Nitric Oxide; ACQ: Asthma Control Questionnaire; OCS: Oral Corticosteroids; ICS: Inhaled Corticosteroids: BDP: Beclometasone Diproprionate

Table S3. Baseline demographics of the whole cohort at initial assessment prior to commencing biologics (n=1,111)

Variable	n	Category	Result
**Time to first Annual Review (Years)**	**1,111**		1.1 (1.0,1.3)
**Time to first Annual Review (Months)**	**1,111**	>=9 & <12 months	352 (31.7%)
		>=12 & <18 months	587 (52.8%)
		>=18 & <=24 months	172 (15.5%)
**Sex**	1,111	Female	667 (60.0%)
**Age At First Assessment (Years)**	1,111		52.0 (41.0,61.0)
**Ethnicity**	1,109	White	966 (87.1%)
		Non-White	143 (12.9%)
**Smoking Status**	1,083	Never	723 (66.8%)
**BMI (kg-m2)**	1,099		29.8 (26.1,34.8)
**BMI ≥30kg-m2**		≥30	540 (49.1%)
**Atopic Disease**	1,111		567 (51.0%)
**Depression or Anxiety**	1,111		129 (11.6%)
**Gastro-oesophageal Reflux**	1,111		224 (20.2%)
**Nasal polyps**	1,111		237 (21.3%)
**OCS bursts (Last Year)**	1,086		5 (3,8)
**Any OCS bursts (Last Year)**	1,086		1,020 (93.9%)
**Frequent exacerbator at baseline**	1,086	≥3 exacerbations in last year	906 (83.4%)
**Invasive Ventilations (Ever)**	1,058		101 (9.5%)
**ED Attendances For asthma (Last Year)**	1,073		0 (0,2)
**Any ED Attendance for asthma (Last Year)**	1,073		435 (40.5%)
**Any Hospital Admissions for asthma (Last Year)**	1,081		431 (39.9%)
**Hospital Admissions for asthma (Last Year)**	1,081	≥2	253 (23.4%)
**Highest Blood Eosinophil Count recorded ($N/10^9L$) †**	1,093		0.70 (0.44,1.10)
**Blood Eosinophil Count ($N/10^9L$)**	1,085		0.40 (0.20,0.65)
**Blood Eosinophil Count ($N/10^9L$)**	1,085	<0.15	219 (20.2%)
		≥0.15 & <0.30	131 (12.1%)
		≥0.30 & ≤0.45	264 (24.3%)
		>0.45	471 (43.4%)
**FeNO (ppb)**	831		43.0 (24.0,75.0)
**FeNO**	831	<20ppb	167 (20.1%)
		≥20 & <50ppb	299 (36.0%)
		≥50ppb	365 (43.9%)
**Composite T2-Group**	811	Eos Low (<0.15) / FeNO Low (<20)	48 (5.9%)
		Eos High (≥0.15) / FeNO Low (<20)	111 (13.7%)
		Eos Low (<0.15) / FeNO High (≥20)	126 (15.5%)
		Eos High (≥0.15) / FeNO High (≥20)	526 (64.9%)
**Clinic FEV_1 (L)**	1,089		2.0 (1.5,2.6)
**Clinic FEV_1 (% Predicted)**	1,077		66.9 (52.1,81.5)
**Clinic FVC (L)**	1,062		3.1 (2.5,3.9)
**Clinic FVC (% Predicted)**	1,026		85.9 (73.4,97.8)
**Clinic FEV_1/FVC**	1,062		63.6 (53.7,72.1)
**ACQ5 Score**	954		3.2 (2.0,4.0)
**Uncontrolled Asthma**	954	**ACQ5≥1.5**	807 (84.6%)
**Maintenance OCS**	1,106		638 (57.7%)
**Maintenance OCS (mg)**	1,102634		10 (8,15)
**Inhaled corticosteroids (ICS)**	1,111		1,103 (99.3%)
**ICS Dose (BDP equivalent-µg)**	1,025		2000 (1600,2000)
**Theophylline**	1,099		299 (27.2%)
**Long-acting β2-agonist (LABA)**	1,094		1,013 (92.6%)
**Long acting anti-muscarinic agent (LAMA)**	1,095		709 (64.7%)
**Leukotriene Receptor Antagonists**	1,059		548 (51.7%)

**Maintenance Macrolides**	1,087		100 (9.2%)
**Biologic type commenced**	981	Anti-IL5	828 (84.4%)
		Anti-IgE	150 (15.3%)
		Anti-IL4 Rα	3 (0.3%)
**ERS-ATS Severe Asthma**	1,111	Yes	1,111 (100.0%)

‡ Median (IQR) or count (%) as appropriate; † highest recorded according to prior medical records. 'Last Year' refers to measures assessed using clinical records in the 12 months prior to initial presentation at the severe asthma centre, other measures were as observed or recorded at initial presentation unless otherwise indicated. *among patients on oral corticosteroids**among patients on inhaled corticosteroids

BMI: Body Mass Index; OCS: Oral Corticosteroid Steroids; ED: Emergency Department; FeNO: Fractional Exhaled Nitric Oxide; ppb: Parts per Billion; FEV_1: Forced Expiratory Volume (1 second); FVC: Forced Vital Capacity; ACQ: Asthma Control Questionnaire: ICS: Inhaled Corticosteroid Steroids: BDP: Beclometasone Dipropionate; SABA: Short-Acting Beta-2 Agonists; LABA: Long-Acting Beta-2 Agonists; LAMA: Long-Acting Muscarinic Antagonist; ERS-ATS: European Respiratory Society-American Thoracic Society

Table S4: Baseline demographics of the primary remission/non-remission‡ cohort (n=830)

Variable	n	Category	Result
**Time to first Annual Review (Years)**	**830**		1.1 (1.0,1.3)
**Time to first Annual Review (Months)**	**830**	>=9 & <12 months	279 (33.6%)
		>=12 & <18 months	430 (51.8%)
		>=18 & <=24 months	121 (14.6%)
**Sex**	830	Female	489 (58.9%)
**Age At First Assessment (Years)**	830		52.0 (42.0,61.0)
**Age at Onset of Symptoms (Years)**	737		22.0 (7.0,41.0)
**Age Of Onset of Symptoms (Years)**	737	≥40	209 (28.4%)
**Duration Of Symptoms From Baseline (Years)**	737		23.0 (11.0,37.0)
**Duration Of Symptoms From Baseline (Years)**		<20	308 (41.8%)
		≥12 & <18	147 (19.9%)
		≥18	282 (38.3%)
**Ethnicit**	828	White	724 (87.4%)
**Smoking Status**	809	Never	539 (66.6%)
**BMI (kg-m2)**	823		30.0 (26.3,34.8)
**BMI (kg-m2)**	823	<25	144 (17.5%)
		≥25 & <30	271 (32.9%)
		≥30	408 (49.6%)
**Atopic Disease**	830		427 (51.4%)
**Depression or Anxiety**	830		88 (10.6%)
**Gastro-oesophageal Reflux**	830		144 (17.3%)
**Nasal polyps**	830		171 (20.6%)
**OCS bursts (Last Year)**	817		5 (3,8)
**Any OCS bursts (Last Year)**	817		762 (93.3%)
**Frequent exacerbator at baseline**	817	≥3 exacerbations in last year	668 (81.8%)
**Invasive Ventilations (Ever)**	798		76 (9.5%)
**ED Attendances For asthma (Last Year)**	807		0 (0,2)
**Any ED Attendance for asthma (Last Year)**	807		293 (36.3%)
**Hospital Admissions for asthma (Last Year)**	814		0 (0,1)
**Any Hospital Admissions for asthma (Last Year)**	814		315 (38.7%)
**Hospital Admissions for asthma (Last Year)**	814	≥2	183 (22.4%)
**Highest Blood Eosinophil Count recorded ($N/10^9L$) †**	821		0.70 (0.45,1.08)
**Blood Eosinophil Count ($N/10^9L$)**	819		0.40 (0.20,0.62)
**Blood Eosinophil Count ($N/10^9L$)**	819	<0.15	175 (21.4%)
		≥0.15 & <0.30	96 (11.7%)
		≥0.30 & ≤0.45	196 (23.9%)
		>0.45	352 (43.0%)
**FeNO (ppb)**	638		43.0 (24.0,74.0)
**FeNO (ppb)**	638	<20ppb	123 (19.3%)
		≥20 & <50ppb	234 (36.7%)
		≥50ppb	281 (44.0%)
**Composite Type 2 status**	627	Eos Low (<0.15) / FeNO Low (<20)	40 (6.4%)
		Eos High (≥0.15) / FeNO Low (<20)	79 (12.6%)
		Eos Low (<0.15) / FeNO High (≥20)	107 (17.1%)
		Eos High (≥0.15) / FeNO High (≥20)	401 (64.0%)
**FEV_1 (L)**	818		2.0 (1.5,2.6)
**FEV_1 (% Predicted)**	811		67.2 (52.5,81.9)
**FVC (L)**	802		3.2 (2.5,3.9)
**FVC (% Predicted)**	772		85.5 (74.1,97.7)
**FEV_1/FVC**	802		64.0 (54.3,72.3)
**ACQ5 Score**	736		3.0 (2.0,4.0)
**Uncontrolled Asthma**	736	ACQ5≥1.5	622 (84.5%)
**EuroQoL Health Scale**	350		60.0 (44.0,73.0)
**EuroQoL Utility**	362		0.7 (0.5,0.9)
**Maintenance OCS**	826		475 (57.5%)

**Maintenance OCS (mg) ***	474		10 (8,15)
**Inhaled corticosteroids (ICS)**	830		827 (99.6%)
**ICS Dose (BDP equivalent-µg) ****	775		2000 (1600,2000)
**Theophylline**	821		216 (26.3%)
**Short- acting β2-agonist (SABA)**	823		785 (95.4%)
**Long-acting β2-agonist (LABA)**	818		763 (93.3%)
**Long acting anti-muscarinic agent (LAMA)**	821		525 (63.9%)
**Leukotriene Receptor Antagonists**	799		407 (50.9%)
**Maintenance Macrolides**	816		69 (8.5%)
**Nebuliser**	819		180 (22.0%)
**ERS-ATS Severe Asthma**	830	Yes	830 (100.0%)

‡ ACQ5 <1.5, no mOCS or OCS bursts, FEV_1 above LLN or <100mls less than pre-biologic FEV

‡ Median (IQR) or count (%) as appropriate; † highest recorded according to prior medical records. 'Last Year' refers to measures assessed using clinical records in the 12 months prior to initial presentation at the severe asthma centre, other measures were as observed or recorded at initial presentation unless otherwise indicated

* among patients on oral corticosteroids

** among patients on inhaled corticosteroids

BMI: Body Mass Index; OCS: Oral Corticosteroid Steroids; ED: Emergency Department; FeNO: Fractional Exhaled Nitric Oxide; ppb: Parts per Billion; FEV_1: Forced Expiratory Volume (1 second); FVC: Forced Vital Capacity; ACQ: Asthma Control Questionnaire: ICS: Inhaled Corticosteroid Steroids: BDP: Beclometasone Dipropionate; SABA: Short-Acting Beta Agonists; LABA: Long-Acting Beta Agonists; LAMA: Long-Acting Muscarinic Antagonist; ERS-ATS: European Respiratory Society-American Thoracic Society

Table S5: Data completeness of the primary remission cohort by remission status‡ (n=830)

	Non remission (81.7% (678))		Remission (18.3% (152))	
**Variable**	**n (%) non-missing**	**n (%) missing**	**n (%) non-missing**	**n (%) missing**
**Time until Annual review (Years)**	678 (100.0%)	0 (0.0%)	152 (100.0%)	0 (0.0%)
**Sex**	678 (100.0%)	0 (0.0%)	152 (100.0%)	0 (0.0%)
**Age At First Assessment (Years)**	678 (100.0%)	0 (0.0%)	152 (100.0%)	0 (0.0%)
**Age at Onset of Symptoms (Years)**	600 (88.5%)	78 (11.5%)	137 (90.1%)	15 (9.9%)
**Duration Of Symptoms From Baseline (Years)**	600 (88.5%)	78 (11.5%)	137 (90.1%)	15 (9.9%)
**Ethnicity**	676 (99.7%)	2 (0.3%)	152 (100.0%)	0 (0.0%)
**Smoking Status**	663 (97.8%)	15 (2.2%)	146 (96.1%)	6 (3.9%)
**BMI (kg-m2)**	671 (99.0%)	7 (1.0%)	152 (100.0%)	0 (0.0%)
**Atopic Disease**	678 (98.0%)	14 (2.0%)	152 (100.0%)	0 (0.0%)
**Depression or Anxiety**	678 (100.0%)	0 (0.0%)	152 (100.0%)	0 (0.0%)
**Gastro-oesophageal Reflux**	678 (100.0%)	0 (0.0%)	152 (100.0%)	0 (0.0%)
**Nasal polyps**	678 (100.0%)	0 (0.0%)	152 (100.0%)	0 (0.0%)
**Rescue Steroids In Last Year**	667 (98.4%)	11 (1.6%)	150 (98.7%)	2 (1.3%)
**Frequent exacerbator at baseline**	667 (98.4%)	11 (1.6%)	150 (98.7%)	2 (1.3%)
**Invasive Ventilations (Ever)**	650 (95.9%)	28 (4.1%)	148 (97.4%)	4 (2.6%)
**A&E Attendances For Asthma In Last Year**	661 (97.5%)	17 (2.5%)	146 (96.1%)	6 (3.9%)
**Hospital Admissions For Asthma In Last Year**	666 (98.2%)	12 (1.8%)	148 (97.4%)	4 (2.6%)
**Highest Blood Eosinophil Count (N/10^{9}L) †**	671 (99.0%)	7 (1.0%)	150 (98.7%)	2 (1.3%)
**Blood Eosinophil Count (N/10^{9}L)**	670 (98.8%)	8 (1.2%)	149 (98.0%)	3 (2.0%)
**FeNO (ppb)**	504 (74.3%)	174 (25.7%)	134 (88.2%)	18 (11.8%)
**Composite T2 status**	496 (73.2%)	182 (26.8%)	131 (86.2%)	21 (13.8%)
**FEV1 (L)**	667 (98.4%)	11 (1.6%)	151 (99.3%)	1 (0.7%)
**FEV1 (% Predicted)**	660 (97.3%)	18 (2.7%)	151 (99.3%)	1 (0.7%)
**FVC (L)**	653 (96.3%)	25 (3.7%)	149 (98.0%)	3 (2.0%)
**FVC (% Predicted)**	629 (92.8%)	49 (7.2%)	143 (94.1%)	9 (5.9%)
**FEV1/FVC**	653 (96.3%)	25 (3.7%)	149 (98.0%)	3 (2.0%)
**ACQ5 Score**	598 (88.2%)	80 (11.8%)	138 (90.8%)	14 (9.2%)
**Puffs of SABA most days (ACQ Q6)**	582 (85.8%)	96 (14.2%)	129 (84.9%)	23 (15.1%)
**EuroQoL Health Scale**	273 (40.3%)	405 (59.7%)	77 (50.7%)	75 (49.3%)
**EuroQoL Utility**	285 (42.0%)	393 (58.0%)	77 (50.7%)	75 (49.3%)
**Maintenance OCS**	674 (99.4%)	4 (0.6%)	152 (100.0%)	0 (0.0%)
**Maintenance OCS (mg) ***	397 (99.7%)	1 (0.3%)	77 (100.0%)	0 (0.0%)
**Inhaled corticosteroids (ICS)**	678 (100.0%)	0 (0.0%)	152 (100.0%)	0 (0.0%)
**ICS Dose (BDP equivalent-µg) ****	632 (93.2%)	46 (6.8%)	143 (94.7%)	8 (5.3%)
**Theophylline**	673 (99.3%)	5 (0.7%)	148 (97.4%)	4 (2.6%)
**Short-acting β2-agonist (SABA)**	673 (99.3%)	5 (0.7%)	150 (98.7%)	2 (1.3%)
**Long-acting β2-agonist (LABA)**	667 (98.4%)	11 (1.6%)	151 (99.3%)	1 (0.7%)
**Long acting anti-muscarinic agent (LAMA)**	670 (98.8%)	8 (1.2%)	151 (99.3%)	1 (0.7%)
**Leukotriene Receptor Antagonists**	653 (96.3%)	25 (3.7%)	146 (96.1%)	6 (3.9%)
**Maintenance Macrolides**	665 (98.1%)	13 (1.9%)	151 (99.3%)	1 (0.7%)
**Nebuliser**	670 (98.8%)	8 (1.2%)	149 (98.0%)	3 (2.0%)
**Biologic commenced**	678 (100.0%)	0 (0.0%)	152 (100.0%)	0 (0.0%)
**Biologic type commenced**	609 (89.8%)	69 (10.2%)	138 (90.8%)	14 (9.2%)
**ERS-ATS Asthma**	678 (100.0%)	0 (0.0%)	152 (100.0%)	0 (0.0%)
**Hospital**	678 (100.0%)	0 (0.0%)	152 (100.0%)	0 (0.0%)

‡ ACQ5 <1.5, no mOCS or OCS bursts, FEV_1 above LLN or <100mls less than pre-biologic FEV_1

† highest recorded according to prior medical records. 'Last Year' refers to measures assessed using clinical records in the 12 months prior to initial presentation at the severe asthma centre, other measures were as observed or recorded at initial presentation unless otherwise indicated
*among patients on OCS
** among patients on inhaled corticosteroids

BMI: Body Mass Index; OCS: Oral Corticosteroid Steroids; ED: Emergency Department; FeNO: Fractional Exhaled Nitric Oxide; ppb: Parts per Billion; FEV_1: Forced Expiratory Volume (1 second); FVC: Forced Vital Capacity; ACQ: Asthma Control Questionnaire: ICS: Inhaled Corticosteroid Steroids: BDP: Beclometasone Dipropionate; SABA: Short-Acting Beta Agonists; LABA: Long-Acting Beta Agonists; LAMA: Long-Acting Muscarinic Antagonist; ERS-ATS: European Respiratory Society-American Thoracic Society

Table S6: Summary statistics for scalar variables in the primary remission cohort by remission status‡ (n=830)

	Non remission (81.7% (678))		Remission (18.3% (152))	
**Variable**	**Mean (SD)**	**Median (IQR)**	**Mean (SD)**	**Median (IQR)**
**Time until Annual review (Years)**	1.2 (0.3)	1.1 (1.0,1.4)	1.1 (0.3)	1.1 (1.0,1.2)
**Age At First Assessment (Years)**	49.7 (14.4)	51.0 (41.0,59.0)	55.2 (13.2)	55.0 (48.0,65.0)
**Age at Onset of Symptoms (Years)**	23.9 (18.8)	20.0 (6.0,39.0)	33.6 (21.4)	32.0 (14.0,52.0)
**Duration Of Symptoms From Baseline (Years)**	25.7 (17.1)	24.5 (12.0,37.0)	21.6 (16.6)	20.0 (8.0,32.0)
**BMI (kg-m2)**	31.4 (7.3)	30.5 (26.6,35.1)	29.2 (7.2)	27.9 (25.4,31.8)
**Rescue Steroids In Last Year**	6.1 (4.1)	5 (3,8)	4.7 (3.1)	4 (3,6)
**A&E Attendances For Asthma (Last Year)**	1.6 (3.9)	0 (0,2)	0.6 (1.8)	0 (0,0)
**Hospital Admissions For Asthma (Last Year)**	1.2 (2.1)	0 (0,1)	0.6 (1.2)	0 (0,1)
**Highest Blood Eosinophil Count (N/10^9L) †**	0.88 (1.11)	0.68 (0.40,1.00)	1.13 (1.25)	0.79 (0.58,1.33)
**Blood Eosinophil Count (N/10^9L)**	0.49 (0.64)	0.39 (0.18,0.60)	0.52 (0.50)	0.46 (0.22,0.70)
**FeNO (ppb)**	55.1 (46.9)	41.0 (22.0,72.0)	64.0 (45.8)	51.0 (35.0,81.0)
**FEV1 (L)**	2.0 (0.8)	2.0 (1.5,2.6)	2.2 (0.9)	2.1 (1.6,2.7)
**FEV1 (% Predicted)**	66.8 (20.7)	67.0 (52.4,81.2)	72.2 (23.5)	68.6 (55.0,88.2)
**FVC (L)**	3.2 (1.0)	3.1 (2.5,3.9)	3.4 (1.1)	3.3 (2.7,4.2)
**FVC (% Predicted)**	85.1 (18.8)	85.2 (73.8,97.0)	89.3 (19.8)	86.7 (75.8,99.4)
**FEV1/FVC**	63.5 (21.3)	63.7 (54.3,72.2)	63.6 (12.9)	64.5 (54.4,73.6)
**EuroQoL Health Scale**	54.6 (20.1)	60.0 (40.0,70.0)	67.4 (19.8)	75.0 (50.0,85.0)
**EuroQoL Utility**	0.6 (0.3)	0.7 (0.5,0.8)	0.8 (0.2)	0.9 (0.8,1.0)
**ACQ5 Score**	3.2 (1.3)	3.2 (2.2,4.2)	2.2 (1.4)	2.0 (1.2,3.4)
**Maintenance OCS (mg) ***	13.5 (9.1)	10 (8,15)	10.0 (5.2)	10 (7,10)
**ICS Dose (BDP equivalent-µg) ****	1961.0 (624.6)	2000 (1600,2000)	2005.8 (767.9)	2000 (1600,2000)

‡ ACQ5 <1.5, no mOCS or OCS bursts, FEV_1 above LLN or <100mls less than pre-biologic FEV_1

† highest recorded according to prior medical records. 'Last Year' refers to measures assessed using clinical records in the 12 months prior to initial presentation at the severe asthma centre, other measures were as observed or recorded at initial presentation unless otherwise indicated

* among patients on OCS

* among patients on inhaled corticosteroids

SD: Standard Deviation; IQR: InterQuartile Range; BMI: Body Mass Index; ED: Emergency Department; FEV_1: Forced Expiratory Volume in 1 second; FVC: Forced Vital Capacity; FeNO: Fractional exhaled Nitric Oxide; ACQ: Asthma Control Questionnaire; OCS: Oral Corticosteroids; ICS: Inhaled Corticosteroids: BDP: Beclometasone Diproprionate

Table S7: Clinical characteristics and outcomes at baseline and after 1 year on biologics

	N	Baseline	Annual Review 1	p-value
**Time to first annual review (years)**	1,111	-	1.1 (1.0,1.3)	-
**BMI (kg/m^2)**	955	30.0 (26.1,35.0)	29.9 (26.1,35.1)	0.011
**Rescue Steroids In Last Year**	1040	5 (3,8)	1 (0,3)	<0.001
**Any Rescue Steroids in Last Year**	1040	975 (93.8%)	656 (63.1%)	<0.001
**Frequent exacerbator (≥3 exacerbations in last year)**	1040	864 (83.1%)	302 (29.0%)	0.002
**ED attendances for asthma (last year)**	1015	0 (0,2)	0 (0,0)	<0.001
**Any ED attendance for asthma (last year)**	1015	397 (39.1%)	139 (13.7%)	<0.001
**Hospital admissions for asthma (last year)**	984	0 (0,1)	0 (0,0)	<0.001
**Any hospital admission for asthma (last year)**	984	395 (40.1%)	140 (14.2%)	<0.001
**Blood eosinophil count (10/^{9}L)**	738	0.37 (0.16,0.61)	0.06 (0.00,0.14)	<0.001
**FEV1 (L)**	941	2.0 (1.5,2.6)	2.2 (1.6,2.7)	<0.001
**FEV1 (% predicted)**	929	67.4 (52.4,81.9)	73.3 (58.7,87.5)	<0.001
**FVC (L)**	870	3.2 (2.5,3.9)	3.2 (2.5,4.0)	0.507
**FVC (% predicted)**	831	86.2 (74.0,98.3)	87.8 (74.5,100.0)	0.065
**FEV1/FVC**	868	63.6 (53.6,72.3)	68.0 (58.7,75.7)	<0.001
**FeNO (ppb)**	567	43.0 (23.0,73.0)	36.0 (16.0,69.0)	<0.001
**ACQ5 Score**	851	3.0 (2.0,4.0)	2.0 (0.8,3.2)	<0.001
**ACQ5≥1.5**	851	721 (84.7%)	514 (60.0%)	<0.001
**ACQ6 Score**	806	3.0 (2.0,4.0)	1.8 (0.8,3.2)	<0.001
**ACQ6 ≥1.5**	806	690 (85.6%)	491 (60.9%)	<0.001
**ACQ7 Score**	806	3.1 (2.2,4.0)	2.1 (1.3,3.3)	<0.001
**ACQ7 ≥1.5**	806	712 (88.3%)	549 (68.1%)	<0.001
**Reported number of SABA puffs most days (ACQ Q6)**	806			
None		96 (11.9%)	203 (25.2%)	<0.001
1-2 puffs		120 (14.5%)	168 (20.8%)	
3-4 puffs		155 (19.2%)	135 (16.8%)	
5-8 puffs		165 (20.5%)	135 (16.8%)	
9-12 puffs		123 (15.3%)	85 (10.6%)	
13-16 puffs		73 (9.1%)	36 (4.5%)	
>16 puffs		74 (9.2%)	44 (5.5%)	
**Maintenance OCS**	1085	625 (57.6%)	517 (47.7%)	<0.001

Median (IQR) or count (%) as appropriate 'Last Year' refers to measures assessed using clinical records in the 12 months prior to either baseline visit to the severe asthma clinic or at annual review, otherwise measures were as observed or recorded at the relevant time-point.

BMI: Body Mass Index; OCS: Oral Corticosteroid Steroids; ED: Emergency Department; FeNO: Fractional Exhaled Nitric Oxide; ppb: Parts per Billion; FEV^1: Forced Expiratory Volume (1 second); FVC: Forced Vital Capacity; ACQ: Asthma Control Questionnaire: SABA: Short-Acting Beta Agonists

Table S8: Baseline pre-biologics characteristics of those who meet remission versus those who do not meet remission at annual review (remission criteria ACQ5 <1.5, no mOCS or OCS bursts, FEV_1 above LLN or <100mls less than pre-biologic FEV_1).

Baseline variable	n	Category	Non-remission	Remission	p-value
	**830**		**81.7% (678)**	**18.3% (152)**	
**Time to first Annual Review (Years)**	**830**		1.1 (1.0,1.4)	1.1 (1.0,1.2)	0.097
**Sex**	830	Female	412 (60.8%)	77 (50.7%)	0.022
		Male	266 (39.2%)	75 (49.3%)	
**Age At First Assessment (Years)**	830		51.0 (41.0,59.0)	55.0 (48.0,65.0)	<0.001
**Age At First Assessment (Years)**	830	18-34	111 (16.4%)	12 (7.9%)	0.005
		35-54	296 (43.7%)	61 (40.1%)	
		>=55	271 (40.0%)	79 (52.0%)	
**Age at Onset of Symptoms (Years)**	737		20.0 (6.0,39.0)	32.0 (14.0,52.0)	<0.001
**Age of Onset of Symptoms (Years)**	737	<12	202 (33.7%)	29 (21.2%)	0.012
		≥12 & <18	57 (9.5%)	12 (8.8%)	
		≥18	341 (56.8%)	96 (70.1%)	
**Age Of Onset of Symptoms (Years)**	737	(≥40)	146 (24.3%)	63 (46.0%)	<0.001
**Duration Of Symptoms From Baseline (Years)**	737		24.5 (12.0,37.0)	20.0 (8.0,32.0)	0.008
**Ethnicity**	828	White	583 (86.2%)	141 (92.8%)	0.028
		Non-White	93 (13.8%)	11 (7.2%)	
**Smoking status**	809	Never	430 (64.9%)	109 (74.7%)	0.023
		Current/Ex-smoker	233 (35.1%)	37 (25.4%)	
**BMI (kg-m2)**	823		30.5 (26.6,35.1)	27.9 (25.4,31.8)	0.001
**BMI (kg-m2)**	823	<25	110 (16.4%)	34 (22.4%)	<0.001
		≥25 & <30	204 (30.4%)	67 (44.1%)	
		≥30	357 (53.2%)	51 (33.6%)	
**Atopic Disease**	830		355 (52.4%)	72 (47.4%)	0.226
**Depression or Anxiety**	830		85 (12.5%)	3 (2.0%)	<0.001
**Gastro-oesophageal Reflux**	830		119 (17.6%)	25 (16.4%)	0.745
**Nasal polyps**	830		123 (18.1%)	48 (31.6%)	<0.001
**OCS bursts (Last Year)**	817		5 (3,8)	4 (3,6)	<0.001
**Any OCS bursts (Last Year)**	817		624 (93.6%)	138 (92.0%)	0.493
**Frequent Exacerbator at baseline**	817	Yes (≥3 in Last Year)	554 (83.1%)	114 (76.0%)	0.043
**Invasive Ventilations (Ever)**	798		68 (10.5%)	8 (5.4%)	0.058
**Any ED Attendance for asthma (Last Year)**	807		259 (39.2%)	34 (23.3%)	<0.001
**Any Hospital Admissions for asthma (Last Year)**	814		270 (40.5%)	45 (30.4%)	0.022
**Highest Blood Eosinophil Count ($N/10^9L$) †**	821		0.68 (0.40,1.00)	0.79 (0.58,1.33)	<0.001
**Highest Blood Eosinophil Count ($N/10^9L$) †**	821	<0.15	13 (1.9%)	4 (2.7%)	0.006
		≥0.15 & <0.3	27 (4.0%)	4 (2.7%)	
		≥0.3 & <0.5	157 (23.4%)	20 (13.3%)	
		≥0.5 & <1.0	292 (43.5%)	61 (40.7%)	
		≥1.0	182 (27.1%)	61 (40.7%)	
**Blood Eosinophil Count ($N/10^9L$)**	819		0.39 (0.18,0.60)	0.46 (0.22,0.70)	0.011
**FeNO (ppb)**	638		41.0 (22.0,72.0)	51.0 (35.0,81.0)	0.002
**FeNO (ppb)**	638	<20	111 (22.0%)	12 (9.0%)	0.001
		≥20 & <50	184 (36.5%)	50 (37.3%)	
		≥50	209 (41.5%)	72 (53.7%)	
**Composite Type 2 status**	627	Eos Low (<0.15) / FeNO Low (<20)	38 (7.7%)	2 (1.5%)	0.004
		Eos High (≥0.15) / FeNO Low (<20)	69 (13.9%)	10 (7.6%)	
		Eos Low (<0.15) / FeNO High (≥20)	86 (17.3%)	21 (16.0%)	
		Eos High ≥0.15) / FeNO High (≥20)	303 (61.1%)	98 (74.8%)	
**FEV_1 (L)**	818		2.0 (1.5,2.6)	2.1 (1.6,2.7)	0.073
**FEV_1 (% Predicted)**	811		67.0 (52.4,81.2)	68.6 (55.0,88.2)	0.027
**FVC (L)**	802		3.1 (2.5,3.9)	3.3 (2.7,4.2)	0.048
**FVC (% Predicted)**	772		85.2 (73.8,97.0)	86.7 (75.8,99.4)	0.065

**FEV_1/FVC**	802		63.7 (54.3,72.2)	64.5 (54.4,73.6)	0.498
**ACQ5 Score**	736		3.2 (2.2,4.2)	2.0 (1.2,3.4)	<0.001
**Uncontrolled Asthma**	711	ACQ5 ≥1.5	531 (88.8%)	91 (65.9%)	<0.001
**EuroQoL Health Scale**	350		60.0 (40.0,70.0)	75.0 (50.0,85.0)	<0.001
**EuroQoL Utility**	362		0.7 (0.5,0.8)	0.9 (0.8,1.0)	<0.001
**Maintenance OCS**	826		398 (59.1%)	77 (50.7%)	0.059
**Maintenance OCS (mg)***	474		10 (8,15)	10 (7,10)	0.001
**Inhaled corticosteroids (ICS)**	830		676 (99.7%)	151 (99.3%)	0.500
**ICS Dose (BDP equivalent-ug)****	775		2000 (1600,2000)	2000 (1600,2000)	0.556
**Theophylline**	821		184 (27.3%)	32 (21.6%)	0.153
**SABA**	823		643 (95.5%)	142 (94.7%)	0.644
**Long-acting β2-agonist (LABA)**	818		619 (92.8%)	144 (95.4%)	0.258
**Long acting anti-muscarinic agent (LAMA)**	821		420 (62.7%)	105 (69.5%)	0.113
**Leukotriene Receptor Antagonists**	799		327 (50.1%)	80 (54.8%)	0.303
**Maintenance Macrolides**	816		60 (9.0%)	9 (6.0%)	0.202
**Nebuliser**	819		164 (24.5%)	16 (10.7%)	<0.001

*among patients on oral corticosteroids

**among patients on inhaled corticosteroids

‡ Median (IQR) or count (%) as appropriate; † highest recorded according to prior medical records. 'Last Year' refers to measures assessed using clinical records in the 12 months prior to initial presentation at the severe asthma centre, other measures were as observed or recorded at initial presentation unless otherwise indicated

OR: Odds Ratio; CI: Confidence Interval; BMI: Body Mass Index; ED: Emergency Department; OCS: Oral Corticosteroid Steroids; FeNO: Fractional Exhaled Nitric Oxide; ppb: Parts per Billion; FEV_1: Forced Expiratory Volume (1 second); FVC: Forced Vital Capacity; ACQ: Asthma Control Questionnaire: SABA: Short-Acting Beta-2 Agonists; ICS: Inhaled Corticosteroid Steroids: LABA: Long-Acting Beta-2 Agonists; LAMA: Long-Acting Muscarinic Antagonist

Table S9: Odds ratios associated with remission‡ for selected covariates (plus 10 unit increase for age and symptom duration, 1 unit increase in BMI)

OR: Odds Ratio; CI: Confidence		Unadjusted model		Adjusted for time to first Annual Review, hospital		Adjusted for time to Annual Review, hospital, baseline ACQ5, baseline exacerbations (ie baseline morbidity)	
**Variable**	**Category**	**N**	**OR (95%CI, p-value)**	**N**	**OR (95%CI, p-value)**	**N**	**OR (95%CI, p-value)**
**Sex**	Male	830	1	830	1	726	1
	Female		0.66 (0.46,0.94), p=0.021		0.64 (0.45,0.93), p=0.018		0.76 (0.51,1.15), p=0.199
**Age at first assessment (years)**	Increase of 10 years	830	1.31 (1.15,1.50), p<0.001	830	1.31 (1.15,1.50), p<0.001	726	1.25 (1.07,1.45), p=0.004
	18-34	830	1	830	1	726	1
	35-54		1.90 (0.99,3.67), p=0.055		1.94 (0.99,3.80), p=0.053		1.94 (0.93,4.03), p=0.077
	>=55		2.69 (1.41,5.15), p=0.003		2.62 (1.35,5.10), p=0.005		2.18 (1.05,4.56), p=0.037
**Duration of symptoms at baseline (years)**	Increase of 10 years	737	0.86 (0.76,0.97), p=0.011	737	0.86 (0.76,0.97), p=0.013	667	0.92 (0.81,1.05), p=0.226
	<10	737	1	737	1	667	1
	10-19		0.49 (0.27,0.87), p=0.015		0.54 (0.29,0.97), p=0.040		0.66 (0.34,1.28), p=0.224
	20-29		0.61 (0.35,1.05), p=0.073		0.64 (0.37,1.14), p=0.128		0.77 (0.41,1.44), p=0.410
	>=30		0.51 (0.32,0.82), p=0.006		0.51 (0.31,0.84), p=0.008		0.70 (0.40,1.22), p=0.210
**Ethnicity**	White	828	1	828	1	724	1
	Non-White		0.51 (0.26,0.98), p=0.043		0.53 (0.26,1.07), p=0.077		0.59 (0.26,1.34), p=0.207
**BMI (kg-m2)**	Increase of 1 kg-m2	823	0.96 (0.93,0.98), p=0.001	823	0.95 (0.92,0.98), p<0.001	7220	0.96 (0.94,0.99), p=0.016
	Not overweight (<25)	823	1	823	1	722	1
	Overweight (>=25 & <30)		1.03 (0.64,1.66), p=0.899		1.00 (0.61,1.63), p=0.992		0.98 (0.56,1.71), p=0.945
	Obese (>=30)		0.46 (0.28,0.74), p=0.002		0.39 (0.24,0.65), p<0.001	722	0.53 (0.30,0.93), p=0.026
**Smoking status**	Never smoker	809	1	809	1	710	1
	Current/ex-smoker		0.61 (0.40,0.91), p=0.017		0.53 (0.34,0.82), p=0.004		0.64 (0.39,1.03), p=0.066
**Depression/anxiety**	No	830	1	830	1	726	1
	Yes		0.13 (0.04,0.42), p=0.001		0.13 (0.04,0.43), p=0.001		0.11 (0.02,0.50), p=0.004
**Composite Type 2 status**	Eos Low (<0.15) / FeNO Low (<20)	627	1	627	1	556	1
	Eos High (>=0.15) / FeNO Low (<20)		2.83 (0.59,13.61), p=0.194		3.26 (0.67,15.93), p=0.144		3.79 (0.73,19.75), p=0.113
	Eos Low (<0.15) / FeNO High (>=20)		4.61 (1.03,20.66), p=0.046		5.33 (1.17,24.34), p=0.031		6.23 (1.28,30.34), p=0.023
	Eos High (>=0.15) / FeNO High (>=20)		6.34 (1.50,26.78), p=0.012		7.44 (1.73,31.95), p=0.007		7.48 (1.64,34.16), p=0.009
**OCS bursts (year prior to biologics)**	0	817	1	817	1	726	1
	1		2.03 (0.81,5.04), p=0.129		1.54 (0.60,3.94), p=0.363		1.44 (0.54,3.87), p=0.465
	2		0.66 (0.25,1.73), p=0.394		0.52 (0.19,1.43), p=0.203		0.57 (0.20,1.65), p=0.302
	>=3		0.70 (0.35,1.37), p=0.292		0.50 (0.24,1.06), p=0.070		0.62 (0.28,1.38), p=0.244
**Maintenance OCS**	No	826	1	826	1	722	1
	Yes		0.74 (0.52,1.05), p=0.092		0.84 (0.57,1.24), p=0.390		0.83 (0.52,1.30), p=0.408
**Biologic Therapy Type**	Anti-IL5	745	1	745	1	674	1
	Anti-IgE		0.22 (0.09,0.55), p=0.001		0.25 (0.10,0.64), p=0.004		0.22 (0.08,0.65), p=0.006
**ACQ5**	<1.5	736	1	736	1	726	1
	>=1.5		0.23 (0.15,0.36), p<0.001		0.19 (0.12,0.31), p<0.001		0.20 0.20 (0.12,0.32), p<0.001

‡ ACQ5 <1.5, no mOCS or OCS bursts, FEV_1 above LLN or <100mls less than pre-biologic FEV_1

Figure S2: Estimated adjusted probability of achieving clinical remission* at the means for selected covariates

‡ ACQ5 <1.5, and no mOCS or OCS bursts and, FEV_1 above LLN or <100mls less than pre-biologic FEV_1 CI: Confidence Interval; BMI: Body Mass Index; Eos: Blood eosinophil count ($N/10^9L$); FeNO: Fractional exhaled Nitric Oxide; OCS: Oral Corticosteroids; mOCS; maintenance OCS; ACQ: Asthma Control Questionnaire; FEV_1; Forced Expiratory Volume (1 second)

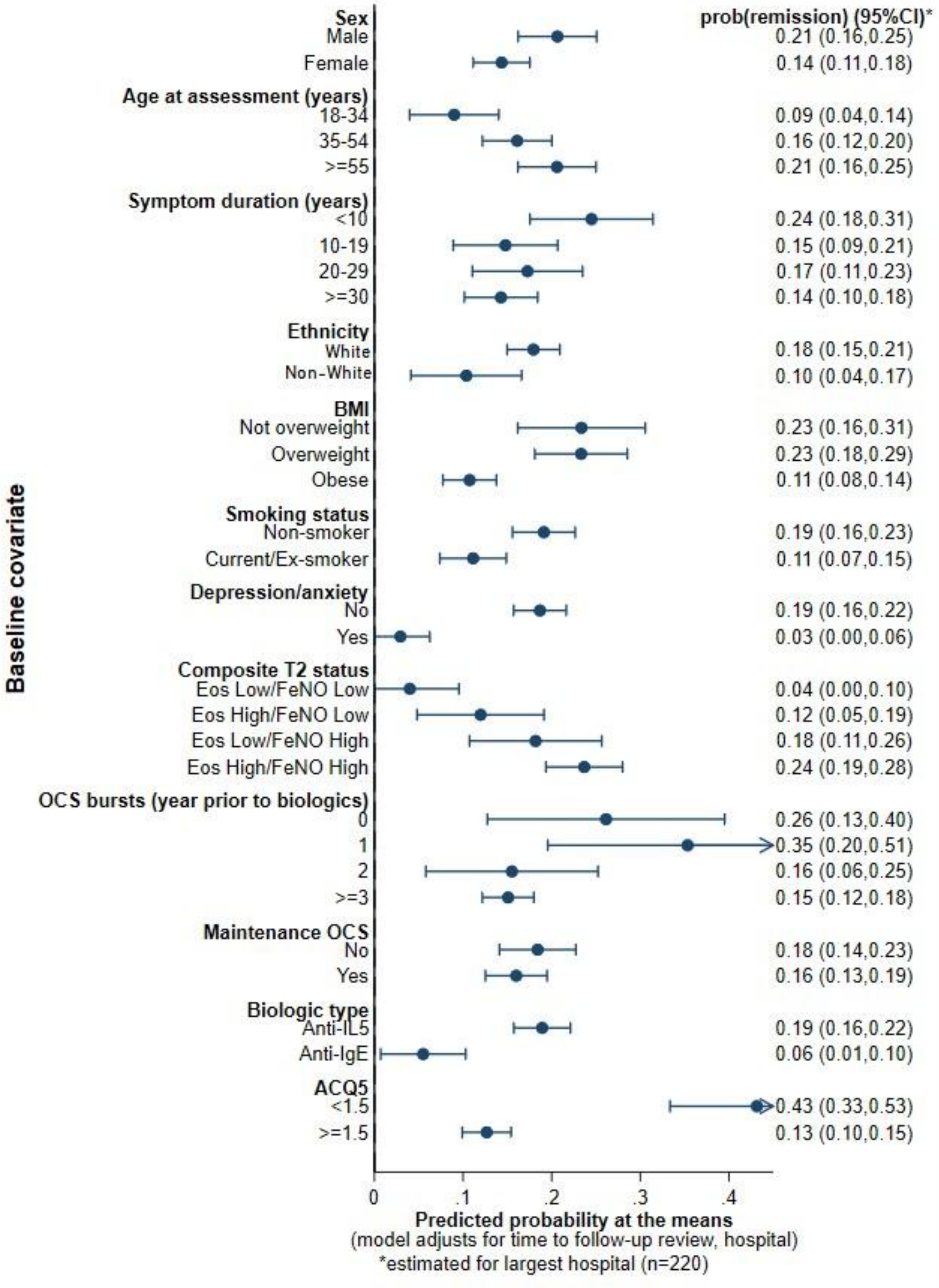
Baseline covariate
prob(remission) (95%CI)*
Sex
Male
Female
0.21 (0.16,0.25)
0.14 (0.11,0.18)
Age at assessment (years)
18-34
35-54
>=55
0.09 (0.04,0.14)
0.16 (0.12,0.20)
0.21 (0.16,0.25)
Symptom duration (years)
<10
10-19
20-29
>=30
0.24 (0.18,0.31)
0.15 (0.09,0.21)
0.17 (0.11,0.23)
0.14 (0.10,0.18)
Ethnicity
White
Non-White
0.18 (0.15,0.21)
0.10 (0.04,0.17)
BMI
Not overweight
Overweight
Obese
0.23 (0.16,0.31)
0.23 (0.18,0.29)
0.11 (0.08,0.14)
Smoking status
Non-smoker
Current/Ex-smoker
0.19 (0.16,0.23)
0.11 (0.07,0.15)
Depression/anxiety
No
Yes
0.19 (0.16,0.22)
0.03 (0.00,0.06)
Composite T2 status
Eos Low/FeNO Low
Eos High/FeNO Low
Eos Low/FeNO High
Eos High/FeNO High
0.04 (0.00,0.10)
0.12 (0.05,0.19)
0.18 (0.11,0.26)
0.24 (0.19,0.28)
OCS bursts (year prior to biologics)
0
1
2
>=3
0.26 (0.13,0.40)
0.35 (0.20,0.51)
0.16 (0.06,0.25)
0.15 (0.12,0.18)
Maintenance OCS
No
Yes
0.18 (0.14,0.23)
0.16 (0.13,0.19)
Biologic type
Anti-IL5
Anti-IgE
0.19 (0.16,0.22)
0.06 (0.01,0.10)
ACQ5
<1.5
>=1.5
0.43 (0.33,0.53)
0.13 (0.10,0.15)
0
.1
.2
.3
.4
Predicted probability at the means
(model adjusts for time to follow-up review, hospital)
*estimated for largest hospital (n=220)

Table S10: Individual area under the curve for each variable included in the regression model.

Variable	Individual AUC (95% CI)
Hospital	0.618 (0.567,0.669)
Time to Follow-up	0.618 (0.567,0.669)
Age At Initial Assessment (Years)	0.655 (0.605,0.706)
Gender	0.644 (0.591,0.696)
Duration Of Symptoms At Baseline (Years)	0.661 (0.614,0.709)
Ethnicity	0.627 (0.576,0.677)
BMI (kg-m2)	0.683 (0.636,0.730)
Smoking Status	0.666 (0.617,0.715)
Depression or Anxiety	0.652 (0.604,0.700)
T2-Group	0.652 (0.600,0.704)
Exacerbations (Last Year)	0.657 (0.615,0.700)
Maintenance OCS	0.630 (0.581,0.678)
Biologic Therapy Type	0.651 (0.603,0.700)
Baseline ACQ5	0.709 (0.661,0.756)

Table S11: Remission criteria ACQ <1.5 AND no OCS bursts or mOCS >5mg/day AND FEV_1 above LLN or <5% from pre-biologics FEV_1: baseline characteristics of those who meet remission at annuak review and those who do not.

Baseline variable	n		Non-remission	Remission	p-value
	**830**		**81.7% (678)**	**18.3% (152)**	
**Time until Annual Review (Years)**	830		1.1 (1.0,1.4)	1.1 (1.0,1.2)	0.067
**Time until Annual Review (Years)**	830	>=9 & <12 months	223 (32.9%)	56 (36.8%)	0.175
		>=12 & <18 months	349 (51.5%)	81 (53.3%)	
		>=18 & <=24 months	106 (15.6%	15 (9.9%)	
**Sex**	830	Female	413 (60.9%)	76 (50.0%)	0.013
**Age At First Assessment (Years)**	830		51.0 (41.0,59.0)	55.0 (48.0,65.0)	<0.001
**Age at Onset of Symptoms (Years)**	737		20.0 (6.0,39.0)	33.5 (14.0,52.0)	<0.001
**Age Of Onset of Symptoms (Years)**	737	(≥40 years)	146 (24.3%)	63 (46.3%)	<0.001
**Duration Of Symptoms From Baseline (Years)**	737		25.0 (12.0,37.0)	20.0 (8.0,31.5)	0.007
**Duration of Symptoms From Baseline (Years)**	737	<20	243 (40.4%)	65 (47.8%)	0.227
		20-29	120 (20.0%)	27 (19.9%)	
		>=30	238 (39.6%)	44 (32.4%)	
**Ethnicity**	828	White	583 (86.2%)	141 (92.8%)	0.028
**Smoking status**	809	Never smoked	430 (64.9%)	109 (74.7%)	0.023
**BMI (kg-m2)**	823		30.5 (26.6,35.2)	27.8 (25.3,31.6)	<0.001
**BMI (kg-m2)**	823	<25	109 (16.2%)	35 (23.0%)	<0.001
		≥25 & <30	204 (30.4%)	67 (44.1%)	
		≥30	358 (53.4%)	50 (32.9%)	
**Atopic Disease**	830		355 (52.4%)	72 (47.4%)	0.226
**Depression or Anxiety**	830		85 (12.5%)	3 (2.0%)	<<0.001
**Gastro-oesophageal Reflux**	830		119 (17.6%)	25 (16.4%)	0.745
**Nasal polyps**	830		124 (18.3%)	47 (30.9%)	0.001
**OCS bursts (Last Year)**	817		5 (3,8)	4 (3,6)	<0.001
**Any OCS bursts (Last Year)**	817		624 (93.6%)	138 (92.0%)	0.493
**Frequent Exacerbator at baseline**	817	Yes (≥3 in Last Year)	554 (83.1%)	114 (76.0%)	0.043
**Invasive Ventilations (Ever)**	798		68 (10.5%)	8 (5.4%)	0.058
**Any ED Attendance for asthma (Last Year)**	807		259 (39.2%)	34 (23.3%)	<0.001
**Any Hospital Admissions for asthma (Last Year)**	814		270 (40.5%)	45 (30.4%)	0.022
**Highest Blood Eosinophil Count (N/10^9L) †**	821		0.68 (0.41,1.00)	0.79 (0.58,1.33)	0.001
**Blood Eosinophil Count (N-109L)**	819		0.40 (0.18,0.60)	0.46 (0.22,0.70)	0.127
**Blood Eosinophil Count (N-109L)**	819	<0.15	148 (22.1%)	27 (18.1%)	0.102
		≥0.15 & <0.30	76 (11.3%)	20 (13.4%)	
		≥0.30 & ≤0.45	169 (25.2%)	27 (18.1%)	
		>0.45	277 (41.3%)	75 (50.3%)	
**FeNO (ppb)**	638		41.0 (22.0,72.0)	51.0 (35.0,81.0)	0.002
**FeNO (ppb)**	638	<20ppb	111 (22.0%)	12 (9.0%)	0.001
		≥20 & <50ppb	185 (36.6%)	49 (36.8%)	
		≥50ppb	209 (41.4%)	72 (54.1%)	
**Composite Type 2 status**	627	Eos Low (<0.15) / FeNO Low (<20)	38 (7.6%)	2 (1.5%)	0.004
		Eos High (≥0.15) / FeNO Low (<20)	69 (13.9%)	10 (7.7%)	
		Eos Low (<0.15) / FeNO High (≥20)	86 (17.3%)	21 (16.2%)	
		Eos High (≥0.15) / FeNO High (≥20)	304 (61.2%)	97 (74.6%)	
**FEV_1 (L)**	818		2.0 (1.5,2.6)	2.1 (1.6,2.7)	0.048
**FEV_1 (% Predicted)**	811		66.9 (52.4,81.2)	68.6 (55.0,88.2)	0.024
**FVC (L)**	802		3.1 (2.5,3.9)	3.3 (2.7,4.2)	0.0129
**FVC (% Predicted)**	772		85.2 (73.6,97.0)	86.7 (76.2,99.4)	0.055
**FEV_1/FVC**	802		63.7 (54.3,72.2)	64.5 (54.4,72.6)	0.530
**ACQ5 Score**	736		3.2 (2.2,4.2)	2.0 (1.2,3.4)	<0.001
**Uncontrolled Asthma**	736	ACQ5 ≥1.5	532 (89.0%)	90 (65.2%)	<0.001
**EuroQoL Health Scale**	350		60.0 (40.0,70.0)	75.0 (50.0,85.0)	<0.001
**EuroQoL Utility**	362		0.7 (0.5,0.8)	0.9 (0.8,1.0)	<0.001

**Maintenance OCS**	826		397 (58.9%)	78 (51.3%)	0.087
**Maintenance OCS (mg) ***	474		10 (8,15)	10 (6,10)	0.001
**Inhaled corticosteroids (ICS)**	830		676 (99.7%)	151 (99.3%)	0.500
**ICS Dose (BDP equivalent-μg) ****	775		2000 (1600,2000)	2000 (1600,2000)	0.619
**Theophylline**	821		184 (27.3%)	32 (21.6%)	0.153
**Short-acting β2-agonist (SABA)**	823		643 (95.5%)	142 (94.7%)	0.644
**Long-acting β2-agonist (LABA)**	818		620 (93.0%)	143 (94.7%)	0.439
**Long acting anti-muscarinic agent (LAMA)**	821		421 (62.8%)	104 (68.9%)	0.163
**Leukotriene Receptor Antagonists**	799		328 (50.2%)	79 (54.1%)	0.397
**Maintenance Macrolides**	816		60 (9.0%)	9 (6.0%)	0.222
**Nebuliser**	819		164 (24.5%)	16 (10.7%)	<0.001

*among patients on oral corticosteroids

**among patients on inhaled corticosteroids

‡ Median (IQR) or count (%) as appropriate; † highest recorded according to prior medical records. 'Last Year' refers to measures assessed using clinical records in the 12 months prior to initial presentation at the severe asthma centre, other measures were as observed or recorded at initial presentation unless otherwise indicated

OR: Odds Ratio; CI: Confidence Interval; BMI: Body Mass Index; ED: Emergency Department; OCS: Oral Corticosteroid Steroids; FeNO: Fractional Exhaled Nitric Oxide; ppb: Parts per Billion; FEV_1: Forced Expiratory Volume (1 second); FVC: Forced Vital Capacity; ACQ: Asthma Control Questionnaire: SABA: Short-Acting Beta Agonists; ICS: Inhaled Corticosteroid Steroids: LABA: Long-Acting Beta Agonists; LAMA: Long-Acting Muscarinic Antagonist

Figure S3: Reasons for not meeting remission criteria after 1 year on biologic therapy. Remission definition: ACQ5 <1.5, no mOCS or OCS bursts, FEV_1 above LLN or ≤5% less than pre-biologic FEV_1

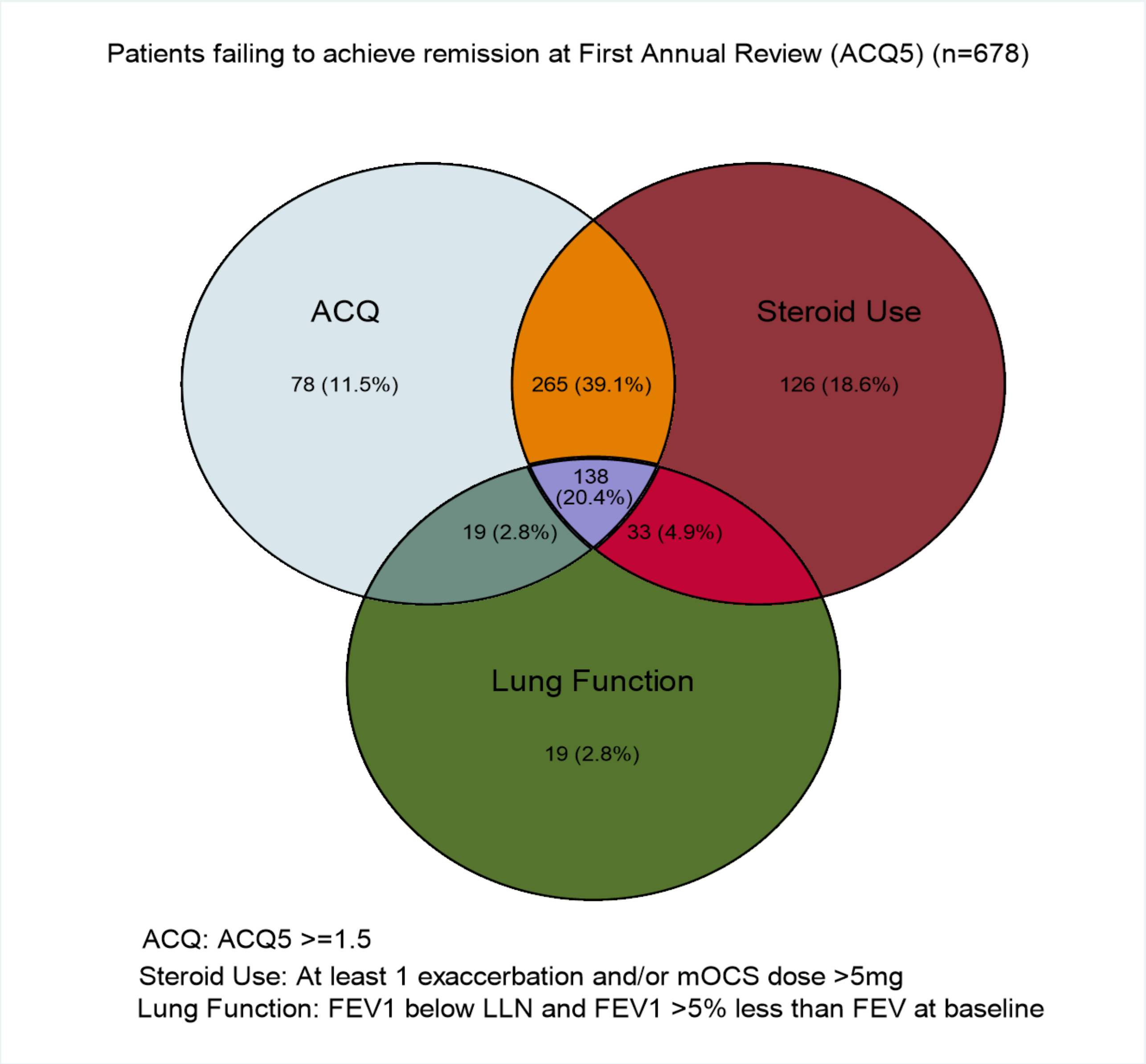

ACQ: Asthma Control Questionnaire; mOCS: maintenance Oral Corticosteroid Steroids: FEV_1; Forced Expiratory Volume (1 second); LLN: Lower Limit of Normal

Table S12: Remission criteria ACQ <1.5 AND no OCS bursts or mOCS >5mg/day AND FEV$_1$ above LLN or no lower than pre-biologics: baseline characteristics of those who meet remission at annual review and those who do not.

	n	Category	Non-remission	Remission	p-value
	**830**		**82.3% (683)**	**17.7% (147)**	
**Time until Annual Review (Years)**	**830**		1.1 (1.0,1.4)	1.1 (1.0,1.2)	0.054
**Time until Annual Review (Years)**	**830**	>=9 & <12 months	225 (32.9%)	54 (36.7%)	0.151
		>=12 & <18 months	351 (51.4%)	79 (53.7%)	
		>=18 & <=24 months	107 (15.7%)	14 (9.5%)	
**Sex**	830	Female	414 (60.6%)	75 (51.0%)	0.032
**Age At First Assessment (Years)**	830		51.0 (41.0,59.0)	55.0 (48.0,65.0)	<0.001
**Age at Onset of Symptoms (Years)**	737		20.0 (6.0,39.0)	33.5 (15.0,52.0)	<0.001
**Age Of Onset of Symptoms (≥40 years)**	737		148 (24.5%)	61 (46.2%)	<0.001
**Duration Of Symptoms From Baseline (Years)**	737		25.0 (12.0,37.0)	20.0 (8.0,31.0)	0.004
**Duration Of Symptoms From Baseline (Years)**	737	0-19	245 (40.5%)	63 (47.7%)	0.210
		20-29	120 (19.8%)	27 (20.5%)	
		>=30	240 (39.7%)	42 (31.8%)	
**Ethnicity**	828	White	587 (86.2%)	137 (93.2%)	0.020
**Smoking status**	809	Never smoked	433 (64.8%)	106 (75.2%)	0.018
**BMI (kg-m2)**	823		30.5 (26.6,35.2)	27.8 (25.3,31.4)	<0.001
**BMI (kg-m2)**	823	<25	111 (16.4%)	33 (22.4%)	<0.001
		≥25 & <30	204 (30.2%)	67 (45.6%)	
		≥30	361 (53.4%)	47 (32.0%)	
**Atopic Disease**	830		358 (52.4%)	69 (46.9%)	0.195
**Depression or Anxiety**	830		86 (12.6%)	2 (1.4%)	<0.001
**Gastro-oesophageal Reflux**	830		120 (17.6%)	24 (16.3%)	0.718
**Nasal polyps**	830		124 (18.2%)	47 (32.0%)	<0.001
**OCS bursts (Last Year)**	817		5 (3,8)	4 (3,6)	<0.001
**Any OCS bursts (Last Year)**	817		629 (93.6%)	133 (91.7%)	0.413
**Frequent Exacerbator at baseline**	817	Yes (≥3 in Last Year)	559 (83.2%)	109 (75.2%)	0.023
**Invasive Ventilations (Ever)**	798		70 (10.7%)	6 (4.2%)	0.017
**Any ED Attendance for asthma (Last Year)**	807		261 (39.2%)	32 (22.7%)	<0.001
**Any Hospital Admissions for asthma (Last Year)**	814		272 (40.5%)	43 (30.1%)	0.020
**Highest Blood Eosinophil Count (N/10^{9}L) †**	821		0.69 (0.41,1.00)	0.79 (0.58,1.33)	<0.001
**Blood Eosinophil Count (N/10^{9}L)**	819		0.39 (0.18,0.60)	0.47 (0.20,0.70)	0.086
**Blood Eosinophil Count (N/10^{9}L)**	819	<0.15	148 (21.9%)	27 (18.8%)	0.049
		≥0.15 & <0.30	78 (11.6%)	18 (12.5%)	
		≥0.30 & ≤0.45	172 (25.5%)	24 (16.7%)	
		>0.45	277 (41.0%)	75 (52.1%)	
**FeNO (ppb)**	638		41.0 (22.0,72.0)	53.0 (36.0,81.0)	<0.001
**FeNO (ppb)**	638	<20ppb	113 (22.2%)	10 (7.8%)	<0.001
		≥20 & <50ppb	186 (36.5%)	48 (37.2%)	
		≥50ppb	210 (41.3%)	71 (55.0%)	
**Composite Type 2 status**	627	Eos Low (<0.15) / FeNO Low (<20)	38 (7.6%)	2 (1.6%)	0.002
		Eos High (≥0.15) / FeNO Low (<20)	71 (14.2%)	8 (6.3%)	
		Eos Low (<0.15) / FeNO High (≥20)	86 (17.2%)	21 (16.7%)	
		Eos High (≥0.15) / FeNO High (≥20)	306 (61.1%)	95 (75.4%)	
**FEV$_1$ (L)**	818		2.0 (1.5,2.6)	2.1 (1.6,2.7)	0.047
**FEV$_1$ (% Predicted)**	811		66.9 (52.4,81.1)	68.7 (55.0,88.9)	0.018
**FVC (L)**	802		3.1 (2.5,3.9)	3.3 (2.7,4.2)	0.038
**FVC (% Predicted)**	772		85.1 (73.8,97.0)	87.1 (76.2,99.4)	0.018
**FEV$_1$/FVC**	802		63.7 (54.3,72.2)	64.6 (55.0,73.7)	0.43
**ACQ5 Score**	736		3.2 (2.2,4.2)	2.0 (1.2,3.2)	<0.001
**Uncontrolled Asthma**	736	ACQ5 ≥1.5	536 (88.9%)	86 (64.7%)	<0.001
**EuroQoL Health Scale**	350		55.0 (40.0,70.0)	75.0 (50.0,85.0)	<0.001
**EuroQoL Utility**	362		0.7 (0.5,0.8)	0.9 (0.8,1.0)	<0.001
**Maintenance OCS**	826		401 (59.1%)	74 (50.3%)	0.053
**Maintenance OCS (mg) ***	474		10 (8,15)	10 (7,10)	0.004

**ICS**	830		681 (99.7%)	146 (99.3%)	0.478
**ICS Dose (BDP equivalent-µg) ****	775		2000 (1600,2000)	2000 (1600,2000)	0.426
**Theophylline**	821		185 (27.3%)	31 (21.7%)	0.166
**Short-acting β2-agonist (SABA)**	823		648 (95.6%)	137 (94.5%)	0.569
**Long-acting β2-agonist (LABA)**	818		624 (92.9%)	139 (95.2%)	0.304
**Long acting anti-muscarinic agent (LAMA)**	821		423 (62.7%)	102 (69.9%)	0.101
**Leukotriene Receptor Antagonists**	799		330 (50.2%)	77 (54.6%)	0.337
**Maintenance Macrolides**	816		61 (9.1%)	8 (5.5%)	0.154
**Nebuliser**	819		166 (24.6%)	14 (9.7%)	<0.001

*among patients on oral corticosteroids**among patients on inhaled corticosteroids[‡] Median (IQR) or count (%) as appropriate; † highest recorded according to prior medical records. 'Last Year' refers to measures assessed using clinical records in the 12 months prior to initial presentation at the severe asthma centre, other measures were as observed or recorded at initial presentation unless otherwise indicated.

OR: Odds Ratio; CI: Confidence Interval; BMI: Body Mass Index; ED: Emergency Department; OCS: Oral Corticosteroid Steroids; FeNO: Fractional Exhaled Nitric Oxide; ppb: Parts per Billion; FEV_1: Forced Expiratory Volume (1 second); FVC: Forced Vital Capacity; ACQ: Asthma Control Questionnaire: SABA: Short-Acting Beta Agonists; ICS: Inhaled Corticosteroid Steroids: LABA: Long-Acting Beta Agonists; LAMA: Long-Acting Muscarinic Antagonist

Figure S4: Reasons for not meeting remission criteria after 1 year on biologic therapy. Remission definition: ACQ5 <1.5, no mOCS or OCS bursts, FEV_1 above LLN or ≤less than pre-biologic FEV1

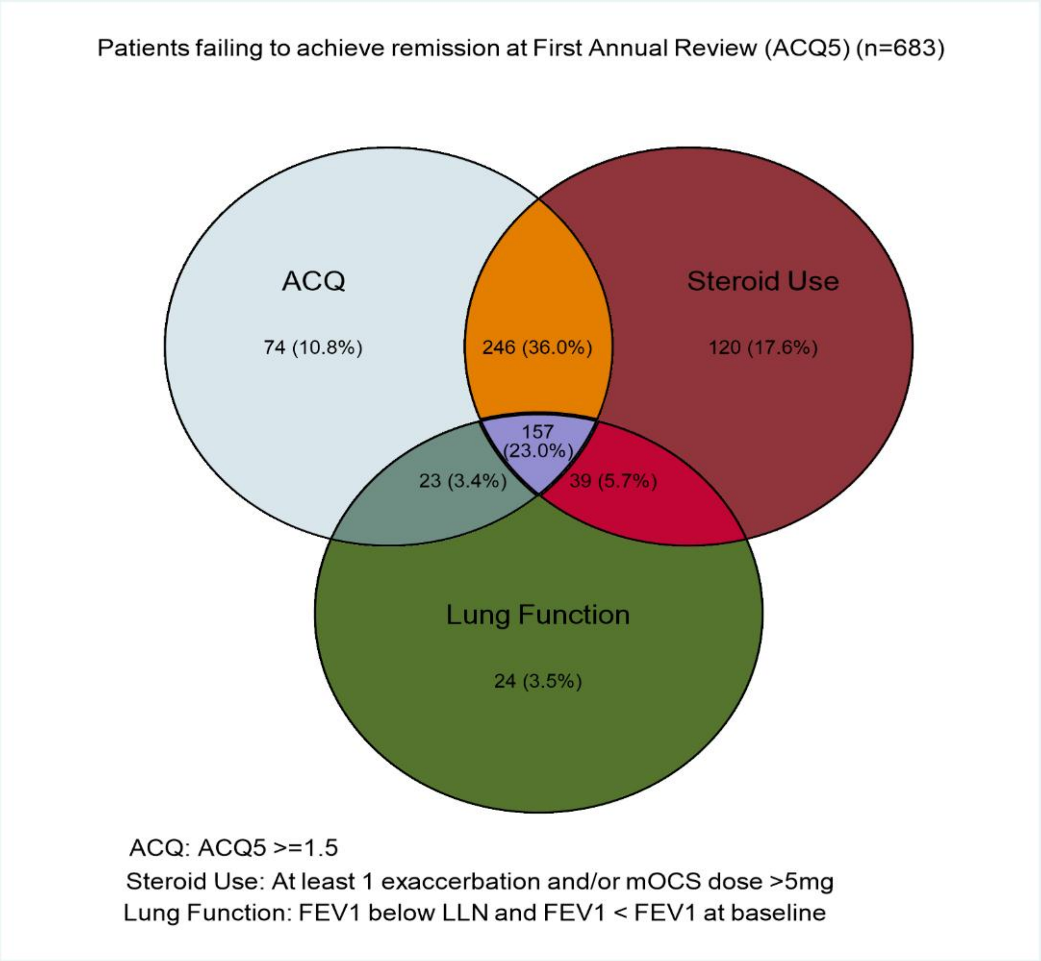

ACQ: Asthma Control Questionnaire; mOCS: maintenance Oral Corticosteroid Steroids: FEV_1; Forced Expiratory Volume (1 second); LLN: Lower Limit of Normal

Table S13: Remission criteria ACQ <1.5 AND no OCS bursts or mOCS >5mg/day: baseline characteristics of those who meet remission at 1 year and those who do not

	n	Category	Non-remission	Remission	p-value
	**925**		**78.8% (729)**	**21.2% (196)**	
**Time until Annual Review (Years)**	925		1.1 (1.0,1.4)	1.1 (1.0,1.3)	0.035
**Sex**	925	Female	445 (61.0%)	100 (51.0%)	0.011
**Age At First Assessment (Years)**	925		51.0 (40.0,59.0)	55.0 (48.0,65.0)	<0.001
**Age at Onset of Symptoms (Years)**	818		20.0 (6.0,38.5)	35.0 (14.0,50.0)	<0.001
**Age Of Onset of Symptoms (≥40 years)**	818		155 (24.1%)	81 (46.6%)	<0.001
**Duration Of Symptoms From Baseline (Years)**	818		25.0 (12.0,38.0)	20.0 (8.0,32.0)	0.003
**Duration of symptoms From Baseline (Years)**	818	0-19	260 (40.4%)	80 (46.0%)	0.175
		20-29	123 (19.1%)	37 (21.3%)	
		>=30	261 (40.5%)	57 (32.8%)	
**Ethnicity**	923	White	623 (85.7%)	178 (90.8%)	0.060
**Smoking status**	903	Never smoked	460 (64.4%)	140 (74.1%)	0.012
**BMI (kg-m2)**	917		30.7 (26.6,35.2)	27.8 (24.9,31.8)	<0.001
**BMI (kg-m2)**	917	<25	119 (16.5%)	50 (25.6%)	<0.001
		≥25 & <30	215 (29.8%)	78 (40.0%)	
		≥30	388 (53.7%)	67 (34.4%)	
**Atopic Disease**	925		382 (52.4%)	92 (46.9%)	0.166
**Depression or Anxiety**	925		100 (13.7%)	5 (2.6%)	<0.001
**Gastro-oesophageal Reflux**	925		138 (18.9%)	34 (17.3%)	0.613
**Nasal polyps**	925		134 (18.4%)	62 (31.6%)	<0.001
**OCS bursts (Last Year)**	908		6 (4,8)	4 (3,6)	<0.001
**Any OCS bursts (Last Year)**	908		672 (94.1%)	178 (91.8%)	0.232
**Frequent Exacerbator at baseline**	908	Yes (≥3 in Last Year)	602 (84.3%)	151 (77.8%)	0.033
**Invasive Ventilations (Ever)**	888		76 (10.9%)	10 (5.2%)	0.019
**Any ED Attendance for asthma (Last Year)**	898		296 (41.7%)	50 (26.6%)	<0.001
**Any Hospital Admission for asthma (Last Year)**	908		299 (41.7%)	58 (30.4%)	0.004
**Highest Blood Eosinophil Count ($N/10^9L$) †**	912		0.68 (0.40,1.00)	0.79 (0.55,1.33)	<0.001
**Blood Eosinophil Count ($N/10^9L$)**	908		0.40 (0.20,0.60)	0.46 (0.21,0.72)	0.082
**Blood Eosinophil Count ($N/10^9L$)**	819	<0.15	148 (20.7%)	37 (19.2%)	0.179
		≥0.15 & <0.30	85 (11.9%)	22 (11.4%)	
		≥0.30 & ≤0.45	181 (25.3%)	37 (19.2%)	
		>0.45	301 (42.1%)	97 (50.3%)	
**FeNO (ppb)**	704		39.0 (22.0,72.0)	51.0 (33.0,80.0)	0.001
**FeNO (ppb)**	704	<20ppb	121 (22.5%)	19 (11.4%)	0.002
		≥20 & <50ppb	198 (36.9%)	59 (35.3%)	
		≥50ppb	218 (40.6%)	89 (53.3%)	
**Composite Type 2 status**	691	Eos Low (<0.15) / FeNO Low (<20)	38 (7.2%)	3 (1.8%)	0.007
		Eos High (≥0.15) / FeNO Low (<20)	79 (15.0%)	15 (9.2%)	
		Eos Low (<0.15) / FeNO High (≥20)	84 (15.9%)	27 (16.6%)	
		Eos High (≥0.15) / FeNO High (≥20)	327 (61.9%)	118 (72.4%)	
**FEV_1 (L)**	907		1.9 (1.5,2.6)	2.1 (1.5,2.7)	0.023
**FEV_1 (% Predicted)**	899		66.4 (51.6,80.6)	70.7 (55.6,87.9)	0.004
**FVC (L)**	887		3.1 (2.5,3.9)	3.2 (2.7,4.2)	0.016
**FVC (% Predicted)**	853		84.6 (72.6,96.7)	86.9 (76.2,99.4)	0.012
**FEV_1/FVC**	887		63.7 (54.3,71.9)	64.5 (56.4,73.4)	0.427
**ACQ5 Score**	822		3.4 (2.2,4.2)	2.2 (1.2,3.2)	<0.001
**Uncontrolled asthma**	822	ACQ5 ≥1.5	579 (89.9%)	117 (65.7%)	<0.001
**EuroQoL Health Scale**	389		55.0 (40.0,70.0)	70.0 (50.0,80.0)	<0.001
**EuroQoL Utility**	404		0.7 (0.5,0.8)	0.9 (0.8,1.0)	<0.001
**Maintenance OCS**	920		422 (58.2%)	101 (51.8%)	0.109
**Maintenance OCS (mg) ***	521		10 (8,15)	10 (6,10)	<0.001
**ICS**	920		422 (58.2%)	101 (51.8%)	0.109
**ICS Dose (BDP equivalent-μg) ****	521		2000 (1600,2000)	2000 (1600,2000)	0.896
**Theophylline**	914		202 (27.9%)	38 (20.1%)	0.031

**Short-acting β2-agonist (SABA)**	918		693 (95.7%)	185 (95.4%)	0.829
**Long-acting β2-agonist (LABA)**	912		668 (93.2%)	183 (93.8%)	0.736
**Long acting anti-muscarinic agent (LAMA)**	916		455 (63.0%)	125 (64.4%)	0.717
**Leukotriene Receptor Antagonists**	888		359 (51.4%)	98 (51.9%)	0.904
**Maintenance Macrolides**	908		70 (9.8%)	12 (6.2%)	0.119
**Nebuliser**	913		179 (24.8%)	23 (12.0%)	<0.001

*among patients on oral corticosteroids**among patients on inhaled corticosteroids‡ Median (IQR) or count (%) as appropriate; † highest recorded according to prior medical records. 'Last Year' refers to measures assessed using clinical records in the 12 months prior to initial presentation at the severe asthma centre, other measures were as observed or recorded at initial presentation unless otherwise indicated.

OR: Odds Ratio; CI: Confidence Interval; BMI: Body Mass Index; ED: Emergency Department; OCS: Oral Corticosteroid Steroids; FeNO: Fractional Exhaled Nitric Oxide; ppb: Parts per Billion; FEV_1: Forced Expiratory Volume (1 second); FVC: Forced Vital Capacity; ACQ: Asthma Control Questionnaire: SABA: Short-Acting Beta Agonists; ICS: Inhaled Corticosteroid Steroids: LABA: Long-Acting Beta Agonists; LAMA: Long-Acting Muscarinic Antagonist

Figure S5: Reasons for not meeting remission criteria after 1 year on biologic therapy. Remission definition: ACQ5 <1.5, no mOCS or OCS bursts

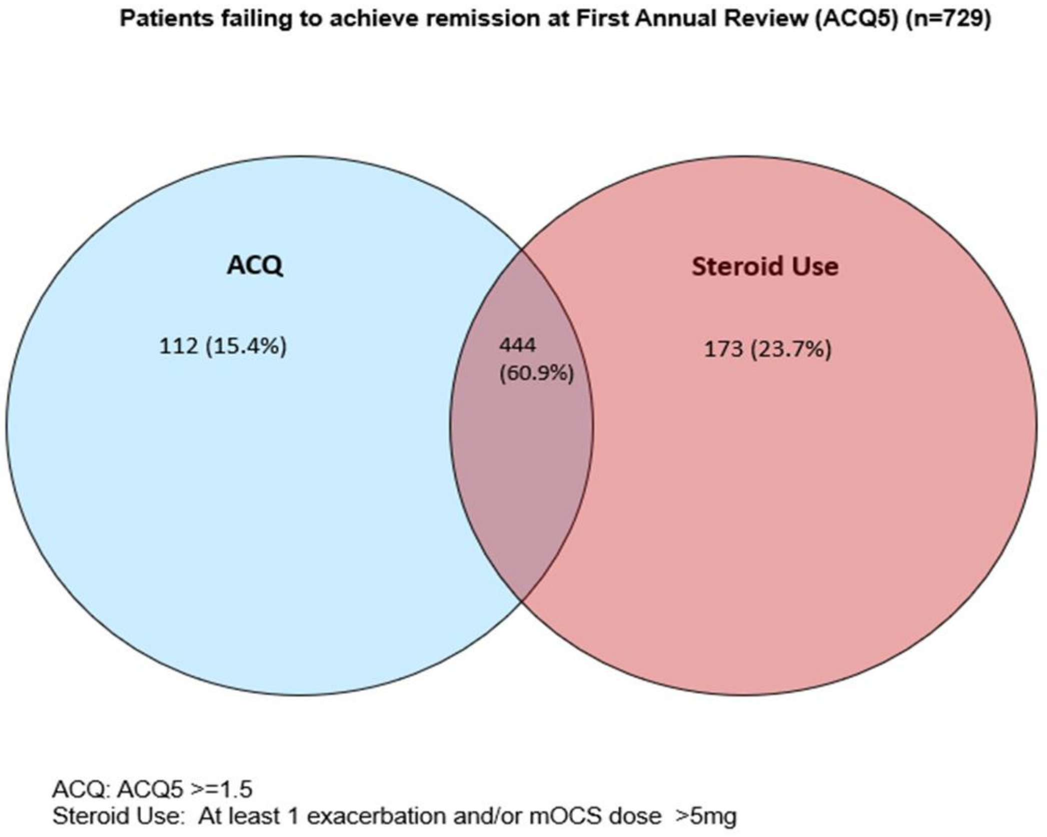

ACQ: Asthma Control Questionnaire; mOCS: maintenance Oral Corticosteroid Steroids

Table S14: Remission criteria ACQ <1.5 AND no OCS bursts or mOCS >5mg/day AND no SABA use: baseline characteristics of those who meet remission at annual review and those who do not

	n	Category	Non-remission	Remission	p-value
	**891**		**86.2% (768)**	**13.8% (123)**	
**Time until Annual Review (Years)**	891		1.1 (1.0,1.4)	1.1 (1.0,1.3)	0.023
**Time until Annual Review (Years)**	891	>=9 & <12 months	226 (29.4%)	49 (39.4%)	0.031
		>=12 & <18 months	409 (53.3%)	61 (49.6%)	
		>=18 & <=24 months	133 (17.3%)	13 (10.6%)	
**Sex**	891	Female	463 (60.3%)	63 (51.2%)	0.058
**Age At First Assessment (Years)**	891		51.0 (41.0,60.0)	55.0 (48.0,65.0)	<0.001
**Age at Onset of Symptoms (Years)**	785		20.0 (6.0,40.0)	40.0 (17.0,53.0)	<0.001
**Age Of Onset of Symptoms (≥40 years)**	785		171 (25.3%)	56 (51.9%)	<0.001
**Duration Of Symptoms From Baseline (Years)**	785		24.0 (12.0,38.0)	19.5 (7.5,31.0)	0.002
**Duration of symptoms From Baseline (Years)**	785	0-19	276 (40.8%)	54 (50.0%)	0.196
		20-29	134 (19.8%)	18 (16.7%)	
		>=30	267 (39.4%)	36 (33.3%)	
**Ethnicity**	889	White	657 (85.8%)	112 (91.1%)	0.111
**Smoking status**	869	Never smoked	485 (64.5%)	87 (74.4%)	0.036
**BMI (kg-m2)**	885		30.5 (26.6,35.2)	26.7 (24.7,30.5)	<0.001
**BMI (kg-m2)**	885	<25	133 (17.4%)	33 (27.0%)	<0.001
		≥25 & <30	227 (29.8%)	54 (44.3%)	
		≥30	403 (52.8%)	35 (28.7%)	
**Atopic Disease**	891		397 (51.7%)	58 (47.2%)	0.436
**Depression or Anxiety**	891		97 (12.6%)	1 (0.8%)	<0.001
**Gastro-oesophageal Reflux**	891		147 (19.1%)	23 (18.7%)	0.908
**Nasal polyps**	891		147 (19.1%)	46 (37.4%)	<0.001
**OCS bursts (Last Year)**	874		6 (3,8)	5 (3,6)	<0.001
**Any OCS bursts (Last Year)**	874		707 (93.9%)	109 (90.1%)	0.118
**Frequent Exacerbator at baseline**	874	Yes (≥3 in Last Year)	631 (83.8%)	95 (78.5%)	0.150
**Invasive Ventilations (Ever)**	855		81 (11.0%)	3 (2.5%)	0.002
**Any ED Attendance for asthma (Last Year)**	864		311 (41.6%)	31 (26.5%)	0.001
**Any Hospital Admission for asthma (Last Year)**	874		312 (41.3%)	35 (29.4%)	0.003
**Highest Blood Eosinophil Count (N/10^9L) †**	878		0.67 (0.40,1.00)	0.90 (0.55,1.41)	<0.001
**Blood Eosinophil Count (N/10^9L)**	874		0.40 (0.20,0.62)	0.44 (0.20,0.78)	0.410
**Blood Eosinophil Count (N/10^9L)**	819	<0.15	149 (19.8%)	26 (21.5%)	0.173
		≥0.15 & <0.30	81 (10.8%)	15 (12.4%)	
		≥0.30 & ≤0.45	195 (25.9%)	20 (16.5%)	
		>0.45	328 (43.6%)	60 (49.6%)	
**FeNO (ppb)**	671		42.0 (23.0,74.0)	50.0 (35.5,75.5)	0.026
**FeNO**	671	<20ppb	120 (21.2%)	11 (10.6%)	0.038
		≥20 & <50ppb	205 (36.2%)	40 (38.5%)	
		≥50ppb	242 (42.7%)	53 (51.0%)	
**Composite Type 2 status**	658	Eos Low (<0.15) / FeNO Low (<20)	33 (5.9%)	2 (2.0%)	0.067
		Eos High (≥0.15) / FeNO Low (<20)	83 (14.9%)	8 (7.9%)	
		Eos Low (<0.15) / FeNO High (≥20)	87 (15.6%)	20 (19.8%)	
		Eos High (≥0.15) / FeNO High (≥20)	354 (63.6%)	71 (70.3%)	
**FEV$_1$ (L)**	873		1.9 (1.4,2.6)	2.2 (1.7,2.8)	0.002
**FEV$_1$ (% Predicted)**	865		65.8 (51.4,79.8)	74.1 (59.1,89.7)	<0.001
**FVC (L)**	853		3.1 (2.5,3.9)	3.3 (2.8,4.3)	0.007
**FVC (% Predicted)**	819		84.3 (72.4,96.7)	87.8 (79.6,100.6)	0.004
**FEV$_1$/FVC**	853		63.5 (53.6,71.7)	65.9 (58.6,74.4)	0.030
**ACQ5 Score**	788		3.2 (2.2,4.0)	1.8 (0.9,2.8)	<0.001
**Uncontrolled Asthma**	788	ACQ5 ≥1.5	604 (89.3%)	65 (58.0%)	<0.001
**EuroQoL Health Scale**	358		55.0 (40.0,70.0)	75.0 (50.0,80.0)	<0.001
**EuroQoL Utility**	375		0.7 (0.5,0.8)	0.9 (0.8,1.0)	<0.001
**Maintenance OCS**	886		444 (58.1%)	59 (48.4%)	0.043
**Maintenance OCS (mg) ***	501		10 (8,15)	10 (6,10)	<0.001
**ICS**	891		764 (99.5%)	122 (99.2%)	0.687
**ICS Dose (BDP equivalent-µg) ****	830		2000 (1600,2000)	2000 (1600,2000)	0.083

**Theophylline**	880		204 (26.7%)	21 (17.9%)	0.042
**Short-acting β2-agonist (SABA)**	884		729 (95.7%)	116 (95.1%)	0.769
**Long-acting β2-agonist (LABA)**	878		705 (93.3%)	112 (91.8%)	0.559
**Long acting anti-muscarinic agent (LAMA)**	882		472 (62.1%)	77 (63.1%)	0.831
**Leukotriene Receptor Antagonists**	854		369 (50.2%)	65 (54.6%)	0.371
**Maintenance Macrolides**	875		69 (9.2%)	6 (5.0%)	0.126
**Nebuliser**	879		173 (22.8%)	9 (7.5%)	<0.001

*among patients on oral corticosteroids**among patients on inhaled corticosteroids‡ Median (IQR) or count (%) as appropriate; † highest recorded according to prior medical records. ‘Last Year’ refers to measures assessed using clinical records in the 12 months prior to initial presentation at the severe asthma centre, other measures were as observed or recorded at initial presentation unless otherwise indicated

OR: Odds Ratio; CI: Confidence Interval; BMI: Body Mass Index; ED: Emergency Department; OCS: Oral Corticosteroid Steroids; FeNO: Fractional Exhaled Nitric Oxide; ppb: Parts per Billion; FEV_1: Forced Expiratory Volume (1 second); FVC: Forced Vital Capacity; ACQ: Asthma Control Questionnaire: SABA: Short-Acting Beta Agonists; ICS: Inhaled Corticosteroid Steroids: LABA: Long-Acting Beta Agonists; LAMA: Long-Acting Muscarinic Antagonist

Figure S6: Reasons for not meeting remission criteria after 1 year on biologic therapy. Remission definition: ACQ5 <1.5, no mOCS or OCS bursts, no daily SABA use

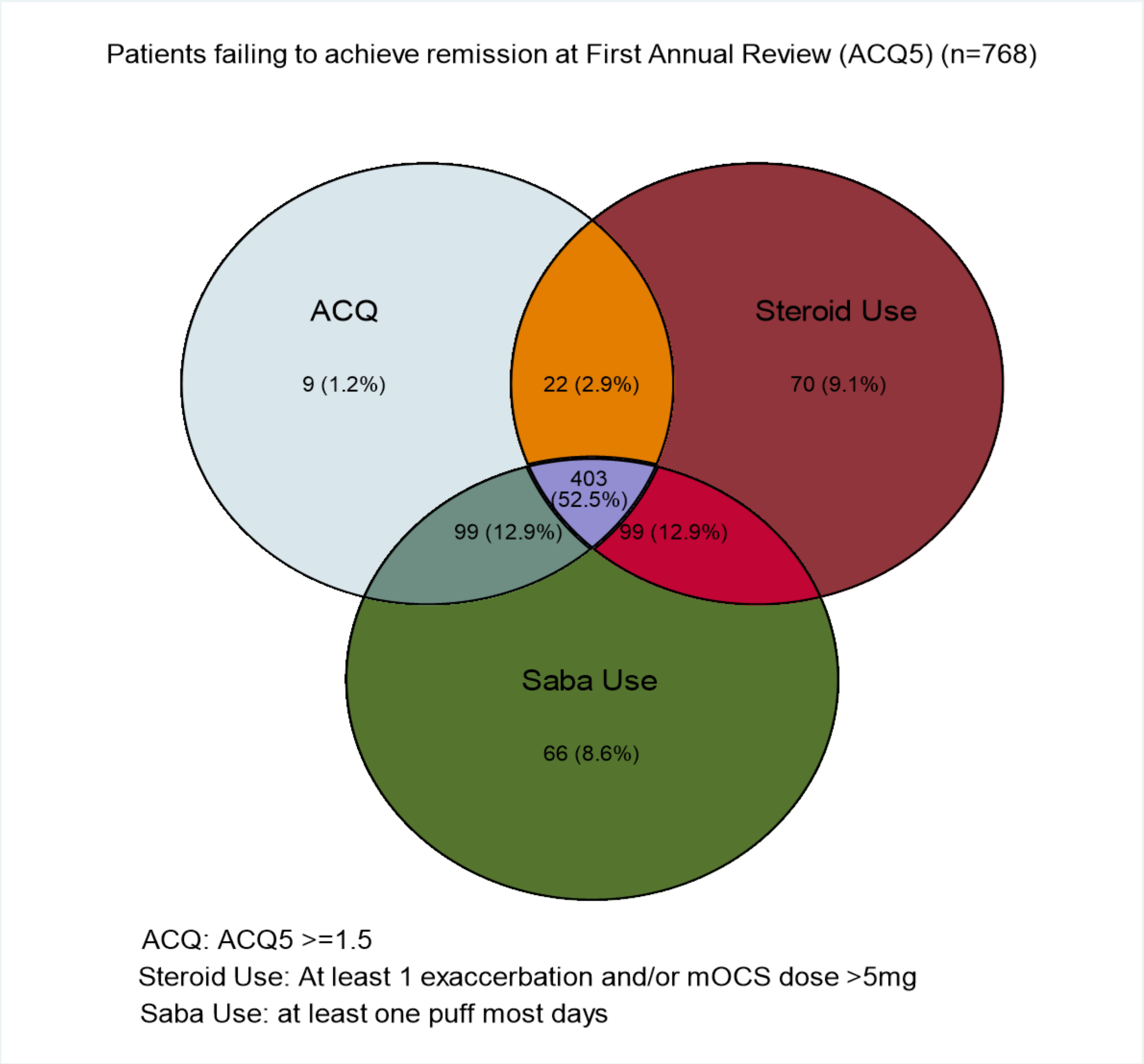

ACQ: Asthma Control Questionnaire; mOCS: maintenance Oral Corticosteroid Steroids; SABA: Short-Acting Beta Agonists

Table S15: Remission criteria ACQ <1.5 AND no OCS bursts or mOCS >5mg/day AND <=2 SABA/day: baseline characteristics of those who meet remission at annual review and those who do not.

	n		Non-remission	Remission	p-value
	**891**		**80.6% (718)**	**19.4% (173)**	
**Time until Annual Review (Years)**	891		1.1 (1.0,1.4)	1.1 (1.0,1.3)	0.020
**Sex**	891	Female	437 (60.9%)	89 (51.4%)	0.024
**Age At First Assessment (Years)**	891		51.0 (40.0,59.0)	56.0 (48.0,65.0)	<0.001
**Age at Onset of Symptoms (Years)**	785		20.0 (5.5,39.0)	36.0 (16.0,52.0)	<0.001
**Age of Onset of Symptoms (Years)**	785	<12	221 (35.0%)	32 (20.9%)	0.002
		≥12 & <18	62 (9.8%)	13 (8.5%)	
		≥18	349 (55.2%)	108 (70.6%)	
**Age Of Onset of Symptoms (≥40 years)**	785		153 (24.2%)	74 (48.4%)	<0.001
**Duration Of Symptoms From Baseline (Years)**	785		24.0 (12.0,38.0)	20.0 (8.0,32.0)	0.005
**Ethnicity**	889	White	613 (85.6%)	156 (90.2%)	0.115
**Smoking status**	869	Never	450 (64.0%)	122 (73.5%)	0.021
**BMI (kg-m2)**	885		30.6 (26.6,35.3)	27.5 (24.9,30.9)	<0.001
**BMI (kg-m2)**	885	<25	122 (17.1%)	44 (25.6%)	<0.001
		≥25 & <30	208 (29.2%)	73 (42.4%)	
		≥30	383 (53.7%)	55 (32.0%)	
**Atopic Disease**	891		377 (52.5%)	78 (45.1%)	0.080
**Depression or Anxiety**	891		95 (13.2%)	3 (1.7%)	<0.001
**Gastro-oesophageal Reflux**	891		138 (19.2%)	32 (18.5%)	0.828
**Nasal polyps**	891		134 (18.7%)	59 (34.1%)	<0.001
**OCS bursts (Last Year)**	874		6 (4,8)	4 (3,6)	<0.001
**Any OCS bursts (Last Year)**	874		661 (94.0%)	155 (90.6%)	0.111
**Frequent Exacerbator at baseline**	874	Yes (≥3 in Last Year)	595 (84.6%)	131 (76.6%)	0.012
**Invasive Ventilations (Ever)**	855		77 (11.2%)	7 (4.2%)	0.006
**Any ED Attendance for asthma (Last Year)**	864		298 (42.6%)	44 (26.7%)	<0.001
**Any Hospital Admission for asthma (Last Year)**	874		300 (42.5%)	47 (28.0%)	<0.001
**Highest Blood Eosinophil Count (N/10^9L) †**	878		0.67 (0.40,1.00)	0.79 (0.55,1.33)	<0.001
**Blood Eosinophil Count (N/10^9L)**	874		0.40 (0.20,0.62)	0.46 (0.20,0.76)	0.128
**Blood Eosinophil Count (N/10^9L)**	819	<0.15	140 (19.9%)	35 (20.5%)	0.155
		≥0.15 & <0.30	79 (11.2%)	17 (9.9%)	
		≥0.30 & ≤0.45	183 (26.0%)	32 (18.7%)	
		>0.45	301 (42.8%)	87 (50.9%)	
**FeNO (ppb)**	671		41.0 (22.0,72.0)	51.0 (35.0,81.0)	0.002
**FeNO (ppb)**	671	<20ppb	116 (22.1%)	15 (10.3%)	0.003
		≥20 & <50ppb	192 (36.6%)	53 (36.3%)	
		≥50ppb	217 (41.3%)	78 (53.4%)	
**Composite Type 2 status**	658	Eos Low (<0.15) / FeNO Low (<20)	32 (6.2%)	3 (2.1%)	0.011
		Eos High (≥0.15) / FeNO Low (<20)	80 (15.5%)	11 (7.7%)	
		Eos Low (<0.15) / FeNO High (≥20)	81 (15.7%)	26 (18.2%)	
		Eos High (≥0.15) / FeNO High (≥20)	322 (62.5%)	103 (72.0%)	
**FEV_1 (L)**	873		1.9 (1.4,2.6)	2.0 (1.6,2.7)	0.031
**FEV_1 (% Predicted)**	865		66.5 (51.5,80.1)	69.0 (55.6,88.0)	0.005
**FVC (L)**	853		3.1 (2.5,3.9)	3.2 (2.7,4.2)	0.035
**FVC (% Predicted)**	819		84.6 (72.9,96.7)	86.8 (75.7,100.0)	0.029
**FEV_1/FVC**	853		63.7 (54.0,71.8)	64.4 (56.5,73.4)	0.49
**ACQ5 Score**	788		3.2 (2.2,4.0)	2.2 (1.0,3.2)	<0.001
**Uncontrolled Asthma**	788	ACQ5 ≥1.5	567 (89.9%)	102 (65.0%)	<0.001
**EuroQoL Health Scale**	358		55.0 (40.0,70.0)	70.0 (50.0,80.0)	<0.001
**EuroQoL Utility**	375		0.7 (0.5,0.8)	0.9 (0.8,1.0)	<0.001
**Maintenance OCS**	886		410 (57.4%)	93 (54.1%)	0.426
**Maintenance OCS (mg) ***	501		10 (8,15)	10 (6,10)	<0.001
**ICS**	830		715 (99.6%)	171 (98.8%)	0.243
**ICS Dose (BDP equivalent-µg) ****	830		2000 (1600,2000)	2000 (1600,2000)	0.671
**Theophylline**	880		191 (26.8%)	34 (20.5%)	0.095
**Short-acting β2-agonist (SABA)**	884		682 (95.7%)	163 (95.3%)	0.850
**Long-acting β2-agonist (LABA)**	878		657 (93.1%)	160 (93.0%)	0.987

**Long acting anti-muscarinic agent (LAMA)**	882		439 (61.7%)	110 (64.3%)	0.532
**Leukotriene Receptor Antagonists**	854		348 (50.7%)	86 (51.5%)	0.845
**Maintenance Macrolides**	875		67 (9.5%)	8 (4.7%)	0.043
**Nebuliser**	879		167 (23.5%)	15 (8.9%)	<0.001

*among patients on oral corticosteroids**among patients on inhaled corticosteroids[‡] Median (IQR) or count (%) as appropriate; † highest recorded according to prior medical records. 'Last Year' refers to measures assessed using clinical records in the 12 months prior to initial presentation at the severe asthma centre, other measures were as observed or recorded at initial presentation unless otherwise indicated

OR: Odds Ratio; CI: Confidence Interval; BMI: Body Mass Index; ED: Emergency Department; OCS: Oral Corticosteroid Steroids; FeNO: Fractional Exhaled Nitric Oxide; ppb: Parts per Billion; FEV_1: Forced Expiratory Volume (1 second); FVC: Forced Vital Capacity; ACQ: Asthma Control Questionnaire: SABA: Short-Acting Beta Agonists; ICS: Inhaled Corticosteroid Steroids: LABA: Long-Acting Beta Agonists; LAMA: Long-Acting Muscarinic Antagonist

Figure S7: Reasons for not meeting remission criteria after 1 year on biologic therapy. Remission definition: ACQ5 <1.5, no mOCS or OCS bursts, ≤2 puffs SABA/day

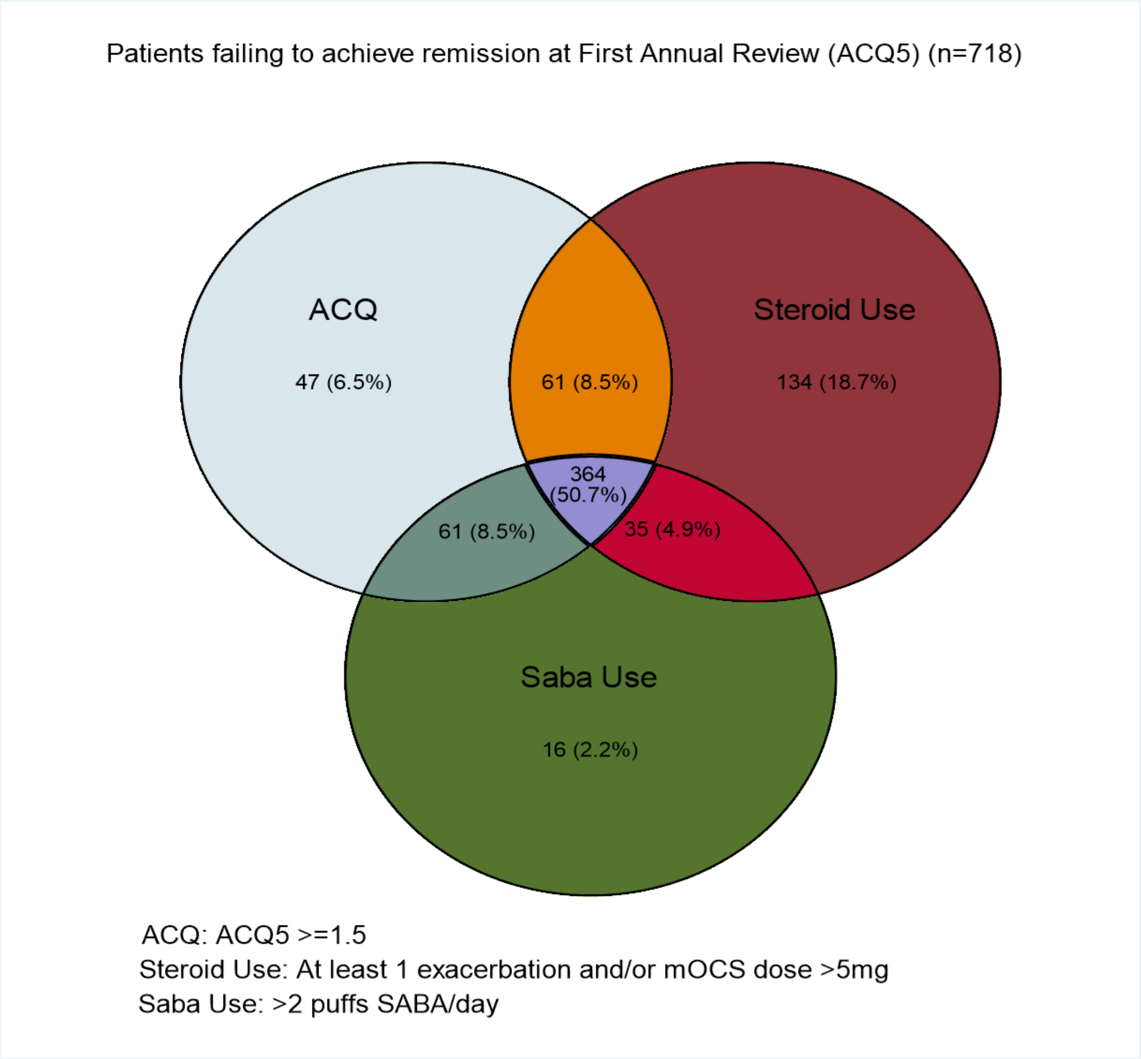

ACQ: Asthma Control Questionnaire; mOCS: maintenance Oral Corticosteroid Steroids; SABA: Short-Acting Beta Agonists

Table S16: Remission criteria ACQ5 <=0.75 AND no OCS bursts or mOCS >5mg/day AND FEV_1 above LLN or <100mls less than pre-biologic FEV_1: baseline characteristics of those who meet remission at 1 year and those who do not

	n		Non-remission	Remission	p-value
	**830**		**88.1% (731)**	**11.9 % (99)**	
**Time until Annual Review (Years)**	830		1.1 (1.0,1.4)	1.1 (1.0,1.2)	0.168
**Sex**		Female	437 (59.8%)	52 (52.5%)	0.168
**Age At First Assessment (Years)**	830		51.0 (41.0.60)	55.0 (47.0,66.0)	0.001
**Age at Onset of Symptoms (Years)**	737		21.0 (6.0,40.0)	31.0 (16.0,52.0)	<0.001
**Duration Of Symptoms From Baseline (Years)**	737		24.0 (11.0,37.0)	20.0 (8.0,33.0)	0.057
**Ethnicity**	828	White	629 (86.3%)	95 (96.0%)	0.006
**Smoking status**	809	Never smoked	468 (65.5%)	71 (74.7%)	0.179
**BMI (kg-m2)**	823		30.3 (26.6,35.1)	27.3 (25.0,31.0)	<0.001
**Atopic Disease**	830		382 (52.3%)	45 (45.5%)	0.202
**Depression or Anxiety**	830		88 (12.0%)	0(0.0%)	<0.001
**Gastro-oesophageal Reflux**	830		128 (17.5%)	16 (16.2%)	0.739
**Nasal polyps**	830		136 (18.6%)	35 (35.4%)	<0.001
**OCS bursts (Last Year)**	817		5 (3,8)	4 (2,6)	0.001
**Any OCS bursts (Last Year)**	817		676 (94.0%)	86 (87.8%)	0.020
**Frequent Exacerbator at baseline**	817	Yes (≥3 in Last Year)	595 (82.8%)	73 (74.5%)	0.047
**Invasive Ventilations (Ever)**	798		0 (0,0)	0 (0,0)	0.059
**Any ED Attendance for asthma (Last Year)**	807		276 (38.8%)	17 (17.9%)	<0.001
**Any Hospital Admissions for asthma (Last Year)**	814		289 (40.3%)	26 (26.8%)	0.010
**Highest Blood Eosinophil Count ($N/10^9L$) †**	821		0.69 (0.43,1.00)	0.90 (0.60,1.41)	<0.001
**Blood Eosinophil Count ($N/10^9L$)**	819		0.39 (0.18,0.60)	0.53 (0.21,0.78)	0.028
**Blood Eosinophil Count ($N/10^9L$)**	819	<0.15	158 (21.9%)	17 (17.5%)	0.018
		≥0.15 & <0.30	82 (11.4%)	14 (14.4%)	
		≥0.30 & ≤0.45	183 (25.3%)	13 (13.4%)	
		>0.45	299 (41.4%)	53 (54.6%)	
**FeNO (ppb)**	638		42.0 (23.0,71.0)	54.0 (38.0,88.0)	0.001
**FeNO (ppb)**	638	<20ppb	118 (21.4%)	5 (5.7%)	0.001
		≥20 & <50ppb	203 (36.8%)	31 (35.6%)	
		≥50ppb	230 (41.7%)	51 (58.6%)	
**Composite Type 2 status**	627	Eos Low (<0.15) / FeNO Low (<20)	40 (7.4%)	0(0.0%)	0.002
		Eos High (≥0.15) / FeNO Low (<20)	74 (13.7%)	5 (5.9%)	
		Eos Low (<0.15) / FeNO High (≥20)	93 (17.2%)	14 (16.5%)	
		Eos High (≥0.15) / FeNO High (≥20)	335 (61.8%)	66 (77.6%)	
**FEV_1 (L)**	818		2.0 (1.4,2.6)	2.2 (1.7,2.8)	0.001
**FEV_1 (% Predicted)**	811		66.5 (52.0,81.1)	74.1 (61.1,89.9)	<0.001
**FVC (L)**	802		3.1 (2.5,3.9)	3.3 (2.8,4.3)	0.014
**FVC (% Predicted)**	772		85.1 (73.4,97.1)	87.2 (79.8,102.9)	0.011
**FEV_1/FVC**	802		63.6 (53.6,72.1)	66.4 (58.1,74.1)	0.072
**ACQ5 Score**	736		3.2 (2.2,4.0)	1.7 (0.7,3.0)	<0.001
**Uncontrolled asthma**	736	ACQ5 ≥1.5	569 (88.4%)	53 (57.6%)	<0.001
**EuroQoL Health Scale**	350		60.0 (40.0,70.0)	80.0 (60.0,85.0)	<0.001
**EuroQoL Utility**	362		0.7 (0.5,0.8)	0.9 (0.8,1.0)	<0.001
**Maintenance OCS**	826		424 (58.3%)	51 (51.5%)	0.199
**Maintenance OCS (mg) ***	474		10 (8,15)	10 (5,10)	0.001
**ICS**	830		729 (99.7%)	98 (99.0%)	0.252
**ICS Dose (BDP equivalent-μg) ****	775		2000 (1600,2000)	2000 (1600,2000)	0.469
**Theophylline**	821		194 (26.8%)	22 (22.9%)	0.422
**Short-acting β2-agonist (SABA)**	823		694 (95.6%)	91 (93.8%)	0.433
**Long-acting β2-agonist (LABA)**	818		670 (93.1%)	93 (94.9%)	0.494
**Long acting anti-muscarinic agent (LAMA)**	821		462 (63.9%)	63 (64.3%)	0.941
**Leukotriene Receptor Antagonists**	799		354 (50.2%)	53 (56.4%)	0.261
**Maintenance Macrolides**	816		62 (8.6%)	7 (7.1%)	0.618
**Nebuliser**	819		170 (23.5%)	10 (10.4%)	0.004

*among patients on oral corticosteroids**among patients on inhaled corticosteroids‡ Median (IQR) or count (%) as appropriate; † highest recorded according to prior medical records. 'Last Year' refers to measures assessed using clinical records in the 12 months prior to initial presentation at the severe asthma centre, other measures were as observed or recorded at initial presentation unless otherwise indicated

OR: Odds Ratio; CI: Confidence Interval; BMI: Body Mass Index; ED: Emergency Department; OCS: Oral Corticosteroid Steroids; FeNO: Fractional Exhaled Nitric Oxide; ppb: Parts per Billion; FEV_1: Forced Expiratory Volume (1 second); FVC: Forced Vital Capacity; ACQ: Asthma Control Questionnaire: SABA: Short-Acting Beta Agonists; ICS: Inhaled Corticosteroid Steroids: LABA: Long-Acting Beta Agonists; LAMA: Long-Acting Muscarinic Antagonist

Figure S8: Reasons for not meeting remission criteria after 1 year on biologic therapy. Remission definition: ACQ5 <=0.75, no mOCS or OCS bursts, FEV_1 above LLN or <100mls less than pre-biologic FEV_1

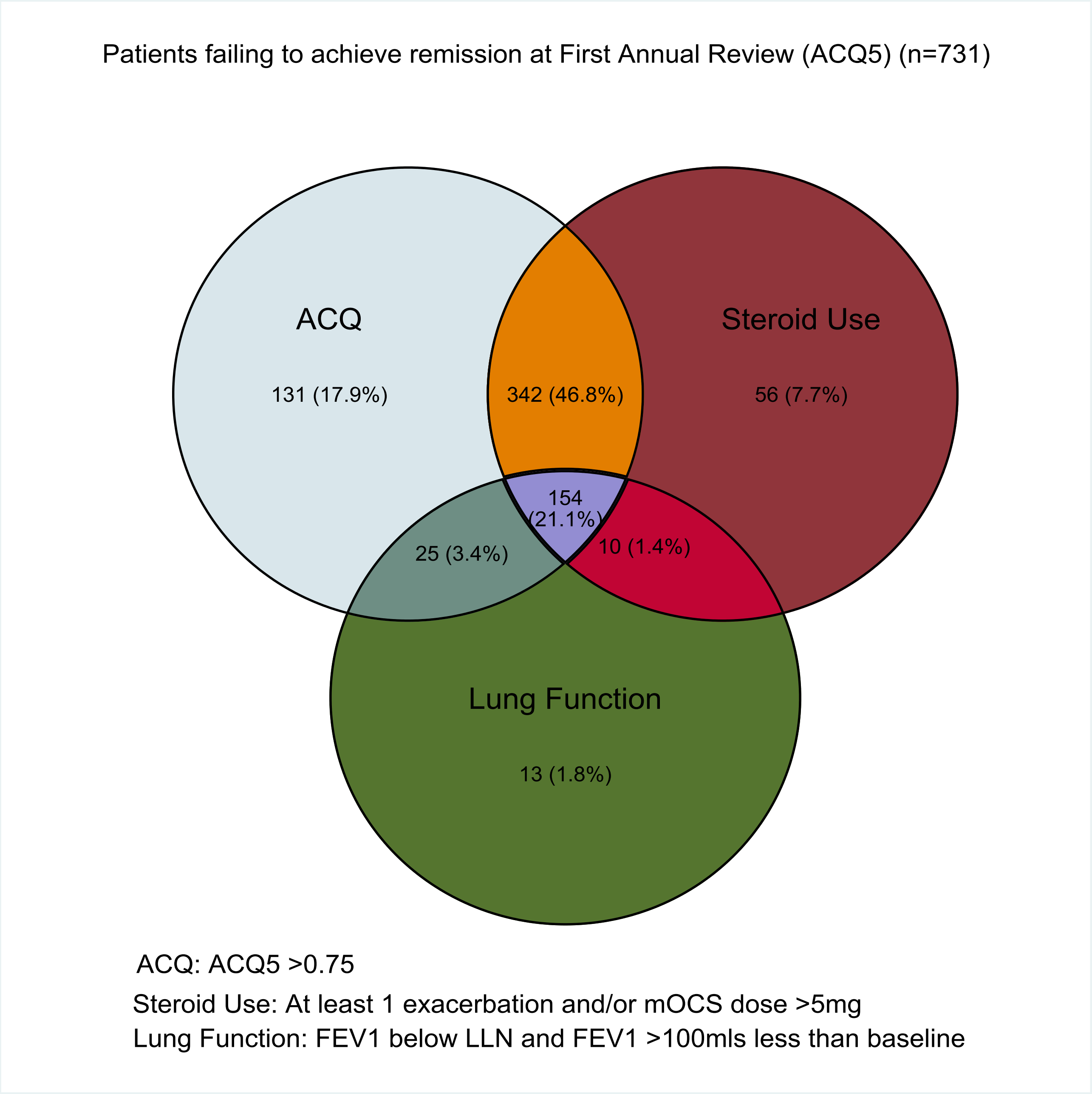

ACQ: Asthma Control Questionnaire; mOCS: maintenance Oral Corticosteroid Steroids: FEV_1; Forced Expiratory Volume (1 second); LLN: Lower Limit of Normal

References online supplement:

E1. Chung FK, Wenzel SE, Brozek JL , Bush A, Castro M, Sterk PJ et al. International ERS/ATS guidelines on definition, evaluation and treatment of severe asthma. Eur Respir J 2014; 43: 343–373

E2. Global initiative for asthma: GINA Difficult to treat and severe asthma, April 2019. URL GINA-Severe-asthma-Pocket-Guide-v2.0-wms-1.pdf (ginasthma.org) (Accessed 08/08/2023)

E3. Menzies-Gow A, Bafadhel M, Busse WW, Casale TB, Kocks JWH, Pavord ID, et al. An expert consensus framework for asthma remission as a treatment goal. Journal of Allergy and Clinical Immunology. 2020 Mar 1;145(3):757–65.

E4. National Institute for Health and Care Excellence (NICE), Guidance for Benralizumab, Accessed 21 April 2023, URL: https://www.nice.org.uk/guidance/TA565/chapter/1-Recommendations

E5. National Institute for Health and Care Excellence (NICE), Guidance for Dupilumab, Accessed 21 April 2023, URL: https://www.nice.org.uk/guidance/TA751/chapter/1-Recommendations

E6. National Institute for Health and Care Excellence (NICE), Guidance for Mepolizumab, Accessed 21 April 2023, URL: https://www.nice.org.uk/guidance/TA671/chapter/1-Recommendations

E7. National Institute for Health and Care Excellence (NICE), Guidance for Omalizumab, Accessed 21 April 2023, URL: https://www.nice.org.uk/guidance/ta278/chapter/1-Guidance

E8. National Institute for Health and Care Excellence (NICE), Guidance for Reslizumab, Accessed 21 April 2023, URL: https://www.nice.org.uk/guidance/TA479/chapter/1-Recommendations

E9. Quanjer PH, Stanojevic S, Cole TJ, Baur X, Hall GL, Culver BH, et al. Multi-ethnic reference values for spirometry for the 3-95-yr age range: The global lung function 2012 equations. European Respiratory Journal. 2012 Dec 1;40(6):1324–43.

E10. Juniper EF, O'Byrne PM, Guyatt GH, Ferrie PJ, King DR. Development and validation of a questionnaire to measure asthma control. Eur Respir J. 1999;14(4):902–7.

E11. Juniper EF, Bousquet J, Abetz L, Bateman ED. Identifying "well-controlled" and "not well-

controlled” asthma using the Asthma Control Questionnaire. Respir Med. 2006 Apr 1;100(4):616–21.

E12. EuroqQol 5 Dimension, 5 level (EQ-5D-5L). Accessed 21 April 2023, URL: https://euroqol.org/eq-5d-instruments/eq-5d-5l-about/
