# Supplementary material for: Clinical remission in severe asthma with biologic therapy: an analysis from the UK Severe Asthma Registry
Source: Eur Respir J. 2023 Dec 14;62(6):2300819. doi: 10.1183/13993003.00819-2023 (PMC10719453; doi:10.1183/13993003.00819-2023)
Supplement: Supplementary file 2 [file ERJ-00819-2023.Shareable.pdf]

SHAREABLE PDF

Clinical remission in severe asthma with biologic therapy: an analysis from the UK Severe Asthma Registry

P. Jane McDowell [1,2], Ron McDowell[3], John Busby[3], M. Chad Eastwood[1,2], Pujan H. Patel[4], David J. Jackson[5], Adel Mansur[6], Mitesh Patel[7], Hassan Burhan[8], Simon Doe[9], Rekha Chaudhuri[10], Robin Gore[11], James W. Dodd [12], Deepak Subramanian[13], Thomas Brown[14] and Liam G. Heaney[1,2] on behalf of the UK Severe Asthma Registry

[1]Wellcome Wolfson Centre for Experimental Medicine, School of Medicine, Dentistry and Biomedical Sciences, Queen's University, Belfast, UK. [2]Belfast Health and Social Care NHS Trust, Belfast, UK. [3]Centre for Public Health, School of Medicine, Dentistry and Biomedical Sciences, Queen's University, Belfast, UK. [4]Royal Brompton and Harefield Hospitals, London, UK. [5]Guy's Severe Asthma Centre, Guy's Hospital, School of Immunology and Microbial Sciences, King's College London, London, UK. [6]University of Birmingham and Heartlands Hospital, Birmingham, UK. [7]Department of Respiratory Medicine, University Hospitals Plymouth NHS Trust, Derriford Hospital, Plymouth, UK. [8]Royal Liverpool University Hospital, Liverpool, UK. [9]The Newcastle upon Tyne Hospitals NHS Foundation Trust, Newcastle upon Tyne, UK. [10]NHS Greater Glasgow and Clyde Health Board, Gartnavel Hospital, Glasgow, UK. [11]Addenbrooke's Hospital, Cambridge University Hospitals NHS Foundation Trust, Cambridge, UK. [12]Academic Respiratory Unit, University of Bristol, Bristol, UK. [13]University Hospitals of Derby and Burton NHS Foundation Trust, Derby, UK. [14]Portsmouth Hospitals NHS Trust, Portsmouth, UK.

Corresponding author: P. Jane McDowell (janemcdowell@doctors.org.uk)

Check for updates

Shareable abstract (@ERSpublications)
Analysis of a real-world severe asthma registry shows clinical remission rates of 18%; associated pre-biologic characteristics include male sex, never smoking, BMI <30 kg·m^{-2}, shorter disease duration, T2-high biomarkers and lower symptom burden https://bit.ly/46JLeDb

Cite this article as: McDowell PJ, McDowell R, Busby J, *et al*. Clinical remission in severe asthma with biologic therapy: an analysis from the UK Severe Asthma Registry. *Eur Respir J* 2023; 62: 2300819 [DOI: 10.1183/13993003.00819-2023].

This extracted version can be shared freely online.

Copyright ©The authors 2023.

This version is distributed under the terms of the Creative Commons Attribution Licence 4.0.

This article has an editorial commentary: https://doi.org/10.1183/13993003.01844-2023

Received: 16 May 2023
Accepted: 5 Oct 2023

Abstract

Background Novel biologic therapies have revolutionised the management of severe asthma with more ambitious treatment aims. Here we analyse the definition of clinical remission as a suggested treatment goal and consider the characteristics associated with clinical remission in a large, real-world severe asthma cohort.

Methods This was a retrospective analysis of severe asthma patients registered in the UK Severe Asthma Registry (UKSAR) who met strict national access criteria for biologics. Patients had a pre-biologics baseline assessment and annual review. The primary definition of clinical remission applied included Asthma Control Questionnaire (ACQ)-5 <1.5 and no oral corticosteroids for disease control and forced expiratory volume in 1 s above lower limit of normal or no more than 100 mL less than baseline.

Results 18.3% of patients achieved the primary definition of remission. The adjusted odds of remission on biologic therapy were 7.44 (95% CI 1.73–31.95)-fold higher in patients with type 2 (T2)-high biomarkers. The adjusted odds of remission were lower in patients who were female (OR 0.61, 95% CI 0.45–0.93), obese (OR 0.49, 95% CI 0.24–0.65) or had ACQ-5 ⩾1.5 (OR 0.19, 95% CI 0.12–0.31) pre-biologic therapy. The likelihood of remission reduced by 14% (95% CI 0.76–0.97) for every 10-year increase in disease duration. 12–21% of the cohort attained clinical remission depending on the definition applied; most of those who did not achieve remission failed to meet multiple criteria.

Conclusions 18.3% of patients achieved the primary definition of clinical remission. Remission was more likely in T2-high biomarker patients with shorter duration of disease and less comorbidity. Further research on the optimum time to commence biologics in severe asthma is required.
